# Pressure-improved Scott–Vogelius type elements

**DOI:** 10.1007/s10092-024-00627-8

**Published:** 2024-12-28

**Authors:** Nis-Erik Bohne, Benedikt Gräßle, Stefan A. Sauter

**Affiliations:** Institut für Mathematik, Universität Zürich, Winterthurerstr 190, 8057 Zürich, Switzerland

**Keywords:** *hp* finite elements, Scott–Vogelius elements, Inf-sup stability, Mass conservation, 65N30, 65N12, 76D07

## Abstract

The Scott–Vogelius element is a popular finite element for the discretization of the Stokes equations which enjoys inf-sup stability and gives divergence-free velocity approximations. However, it is well known that the convergence rates for the discrete pressure deteriorate in the presence of certain *critical vertices* in a triangulation of the domain. Modifications of the Scott–Vogelius element such as the recently introduced pressure-wired Stokes element also suffer from this effect. In this paper we introduce a simple modification strategy for these pressure spaces that preserves the inf-sup stability while the pressure converges at an optimal rate.

## Introduction

In this paper we consider the numerical solution of the stationary Stokes equations by conforming Galerkin finite element methods. This is a vivid research topic since many decades in numerical analysis and scientific computing. The unknowns are the vector-valued velocity field and the scalar pressure and the Galerkin discretization is based on the choice of a pair of finite element spaces: one, say ${\textbf{S}}$, for the velocity and one, say *M*, for the pressure approximation. It is well-known that the most *intuitive* choice for ${\textbf{S}}$, i.e., continuous, piecewise polynomials of degree *k* and for *M*, i.e., discontinuous, piecewise polynomials of degree $k-1$ (which we will denote as the full pressure space) can be unstable: although the continuous problem is well-posed the Galerkin discretization may result in a singular system matrix and is not solvable (see, e.g., [24] and [7, Chap. 7] for quadrilateral meshes).

This problem motivated the development of many pairs $\left( {\textbf{S}},M\right)$ of Stokes elements; standard strategies include enrichment of the intuitive velocity space, see, e.g., [16], reducing the intuitive pressure space, see, e.g., [5, 15, 21, 24] and combinations thereof [2, 3, 11, 7, Chap. 3, §7]. Other approaches are based on a consistent modification of the discrete variational formulation and we refer to the overviews [6, 8, 10] for detailed expositions. In any case, a “good” Stokes discretization should have the following features: (a) discrete stability in the form of a discrete inf-sup condition, (b) the divergence of the discrete velocity is zero or very small, (c) the Stokes element $\left( {\textbf{S}},M\right)$ enjoys good approximation properties for the continuous solution depending on its regularity, (d) the element is simple and easy to be implemented.

The Scott–Vogelius element (see [21, 24]) is a very popular element which is based on an appropriate reduction of the full pressure space in the intuitive element described above. The element is inf-sup stable, the discrete velocity is divergence-free, and its implementation very simple. However, it suffers from two drawbacks; (a) the discrete inf-sup constant is not mesh-robust: if some vertex becomes nearly singular – a geometric notion which will be recalled in the paper and which is not related to shape regularity – the discrete inf-sup constant tends to zero and the discretization becomes increasingly ill-posed; (b) in the presence of super-critical vertices (defined in (3.13) below), the approximation property of the pressure space becomes sub-optimal and affects the accuracy of the discrete pressure significantly. As a remedy for drawback (a), mesh refinement strategies are proposed in the literature (see [2, Rem. 2]) or, alternatively, a very simple modification of the Scott–Vogelius element which circumvents mesh refinement is introduced in [13, Def. 3] and called the *pressure-wired Stokes element*. For both methods, the discrete inf-sup constant becomes mesh-robust while the divergence of the discrete velocity for the pressure-wired Stokes element is not exactly zero but small (without any regularity requirement on the solution) and controlled by a parameter $\eta$.

Drawback (b) affects both, the Scott–Vogelius and the pressure-wired Stokes element: if the mesh contains super-critical vertices, a geometric notion which will be introduced in this paper, the discrete pressure converges only at a very sub-optimal rate. In this paper, we propose a method to modify the pressure space so that the pressure converges with optimal rate. The method is very simple: for both, the Scott–Vogelius and the pressure-wired Stokes element, the reduction of the full pressure space in the most intuitive Stokes element is formulated via a linear (local) constraint at each critical vertex written in the form $A_{{\mathcal {T}},{\textbf{z}}}\left( q\right) =0$ for a discrete pressure function *q* and a critical point ${\textbf{z}}$ in the mesh ${\mathcal {T}}$. This condition is replaced at super-critical vertices by another linear side condition which we introduce in this paper. Its definition relies on the explicit knowledge of a local basis (set of *critical functions*) for the orthogonal complement of the reduced pressure space in the full pressure space. We define a linear injection of the reduced pressure space into the full pressure space by adding a linear combination of those critical functions related to super-critical vertices. The coefficients in this linear combination are given by local linear functionals applied to a pressure in the reduced pressure space. We emphasize that algorithmically this modification is simply realized by replacing the linear constraint $A_{{\mathcal {T}},{\textbf{z}}}\left( q\right) =0$ at super-critical vertices with another local linear constraint and hence the computational complexity and the dimension of the pressure space stay unchanged. It turns out that the inf-sup stability and the smallness of the divergence of the discrete velocity is inherited by the resulting element with this modified pressure space. In addition, the modified pressure space satisfies optimal approximation properties.

The paper is organized as follows. We introduce the Stokes problem, its variational form, and the Galerkin discretization in Sect. 2. Section 3 is devoted to the Scott–Vogelius element with the modified pressure space. We introduce the linear functional which serves as the side constraint for the reduction of the intuitive pressure space, prove that the inf-sup stability of the Scott–Vogelius element is inherited, and the divergence of the discrete velocity remains zero. This modification is particularly simple for the Scott–Vogelius element since it can be realized as a postprocessing step applied to the original Scott–Vogelius solution. In Sects. 3.4 and 3.5 we prove that this postprocessed pressure converges at optimal rate. In Sect. 4 we introduce the pressure-improvement strategy for the pressure-wired Stokes element and define the modified pressure space. In contrast to the Scott–Vogelius element, the discretization of the Stokes equation employs the modified pressure space and directly yields the final discrete solution. We prove in Sect. 4.1 that the inf-sup stability of the original pressure-wired Stokes element is inherited to its modified version. In Sect. 4.2 we will show that our pressure-improvement strategy applied to the pressure-wired Stokes element leads to a pressure space with optimal approximation properties. This result is applied to the modified element in Sect. 3.5 and optimal convergence rates for the discrete solution are shown. It remains to investigate the divergence of the corresponding discrete velocity which is considered in Sect. 4.3. The key role is played by the derivation of an explicit basis representation for the orthogonal complement of the modified pressure space (Lemma 4.11). This is used in Theorem 4.10 to prove that the smallness of the velocity divergence is controlled by the parameter $\eta$. For this result, no regularity assumption on the exact solution is imposed. However, if the continuous solution has some regularity, we derive additional convergence rates for the smallness of the divergence.

## The Stokes problem and its numerical discretization

Let $\Omega \subset {\mathbb {R}}^{2}$ be a bounded Lipschitz domain with polygonal boundary $\partial \Omega$. We consider the numerical solution of the Stokes equation

$$\begin{aligned} \begin{array}{lll} -\Delta {\textbf{u}}+\nabla p &  ={\textbf{f}} &  \text {in }\Omega ,\\ \text {div}{\textbf{u}} &  =0 &  \text {in }\Omega \end{array} \end{aligned}$$

with homogeneous Dirichlet boundary conditions for the velocity and the usual normalization condition for the pressure, namely

$$\begin{aligned} {\textbf{u}}={\textbf{0}}\quad \text {on }\partial \Omega \quad \text {and\quad } \int _{\Omega }p=0. \end{aligned}$$

Throughout this paper, standard notation applies for real-valued Lebesgue and Sobolev spaces. Let $H_{0}^{1}\left( \Omega \right)$ be the closure of the space of infinitely smooth, compactly supported functions with respect to the $H^{1}\left( \Omega \right)$ norm. Its dual space is given by $H^{-1}\left( \Omega \right) :=H_{0}^{1}\left( \Omega \right) ^{\prime }$. The scalar product and norm in $L^{2}\left( \Omega \right)$ are written as

$$\begin{aligned} \left( u,v\right) _{L^{2}\left( \Omega \right) }:=\int _{\Omega } uv\quad \text {and}\quad \left\| u\right\| _{L^{2}\left( \Omega \right) }:=\left( u,u\right) _{L^{2}\left( \Omega \right) }^{1/2}. \end{aligned}$$

Vector-valued and $2\times 2$ tensor-valued analogues of these function spaces are denoted by bold and blackboard bold letters, e.g., ${\textbf{H}}^{s}\left( \Omega \right) =\left( H^{s}\left( \Omega \right) \right) ^{2}$ and ${\mathbb {H}}^{s}\left( \Omega \right) =\left( H^{s}\left( \Omega \right) \right) ^{2\times 2}$ and analogously for other quantities.

The ${\textbf{L}}^{2}\left( \Omega \right)$ scalar product and norm for vector-valued functions are given by

$$\begin{aligned} \left( {\textbf{u}},{\textbf{v}}\right) _{{\textbf{L}}^{2}\left( \Omega \right) }:=\int _{\Omega }\left\langle {\textbf{u}},{\textbf{v}}\right\rangle \quad \text {and\quad }\left\| {\textbf{u}}\right\| _{{\textbf{L}}^{2}\left( \Omega \right) }:=\left( {\textbf{u}},{\textbf{u}}\right) _{{\textbf{L}} ^{2}\left( \Omega \right) }^{1/2}, \end{aligned}$$

with the standard Euclidean scalar product $\left\langle \cdot ,\cdot \right\rangle$. In a similar fashion, we define the scalar product and norm in ${\mathbb {L}}^{2}\left( \Omega \right)$ by

$$\begin{aligned} \left( {\textbf{G}},{\textbf{H}}\right) _{{\mathbb {L}}^{2}\left( \Omega \right) }:=\int _{\Omega }\left\langle {\textbf{G}},{\textbf{H}}\right\rangle \quad \text {and\quad }\left\| {\textbf{G}}\right\| _{{\mathbb {L}}^{2}\left( \Omega \right) }:=\left( {\textbf{G}},{\textbf{G}}\right) _{{\mathbb {L}} ^{2}\left( \Omega \right) }^{1/2} \quad \forall {\textbf{G}},{\textbf{H}} \in {\mathbb {L}}^{2}\left( \Omega \right) , \end{aligned}$$

where $\left\langle {\textbf{G}},{\textbf{H}}\right\rangle =\sum _{i,j=1} ^{2}G_{i,j}H_{i,j}$. Finally, let $L_{0}^{2}\left( \Omega \right) :=\left\{ u\in L^{2}\left( \Omega \right) :\int _{\Omega }u=0\right\}$. We introduce the bilinear forms $a:{\textbf{H}}^{1}\left( \Omega \right) \times {\textbf{H}}^{1}\left( \Omega \right) \rightarrow {\mathbb {R}}$ and $b:{\textbf{H}} ^{1}\left( \Omega \right) \times L^{2}\left( \Omega \right) \rightarrow {\mathbb {R}}$ by

2.1 $$\begin{aligned} \begin{array}{ccc} a\left( {\textbf{u}},{\textbf{v}}\right) :=\left( \nabla {\textbf{u}},\nabla {\textbf{v}}\right) _{{\mathbb {L}}^{2}\left( \Omega \right) }&\text { and }&b\left( {\textbf{u}},p\right) =\left( {\text {div}} {\textbf{u}},p\right) _{L^{2}\left( \Omega \right) } \end{array} \end{aligned}$$

with the derivative $\nabla {\textbf{v}}$ and the divergence ${\text {div}} {\textbf{v}}$ of any ${\textbf{v}} \in {\textbf{H}}^{1} \left( \Omega \right)$. Given a source ${\textbf{F}}\in {\textbf{H}}^{-1}\left( \Omega \right)$, the variational form of the stationary Stokes problem seeks $\left( {\textbf{u}},p\right) \in {\textbf{H}}_{0}^{1}\left( \Omega \right) \times L_{0}^{2}\left( \Omega \right)$ such that

2.2 $$\begin{aligned} \begin{array}{lll} a\left( {\textbf{u}},{\textbf{v}}\right) -b\left( {\textbf{v}},p\right) &  ={\textbf{F}}\left( {\textbf{v}}\right) &  \forall {\textbf{v}}\in {\textbf{H}}_{0} ^{1}\left( \Omega \right) ,\\ b\left( {\textbf{u}},q\right) &  =0 &  \forall q\in L_{0}^{2}\left( \Omega \right) . \end{array} \end{aligned}$$

Concerning the well-posedenss of (2.2), we refer, e.g., to [12] for details. In this paper, we consider a conforming Galerkin discretization of (2.2) by a pair $\left( {\textbf{S}},M\right)$ of finite dimensional subspaces of the continuous solution spaces $\left( {\textbf{H}}_{0}^{1}\left( \Omega \right) ,L_{0}^{2}\left( \Omega \right) \right)$. For any given ${\textbf{F}}\in {\textbf{H}}^{-1}\left( \Omega \right)$, the weak formulation seeks $\left( {\textbf{u}}_{{\textbf{S}} },p_{M}\right) \in {\textbf{S}}\times M\;$such that

2.3 $$\begin{aligned} \begin{array}{lll} a\left( {\textbf{u}}_{{\textbf{S}}},{\textbf{v}}\right) -b\left( {\textbf{v}},p_{M}\right) &  ={\textbf{F}}\left( {\textbf{v}}\right) &  \forall {\textbf{v}} \in {\textbf{S}},\\ b\left( {\textbf{u}}_{{\textbf{S}}},q\right) &  =0 &  \forall q\in M. \end{array} \end{aligned}$$

It is well known that the bilinear form $a\left( \cdot ,\cdot \right)$ is symmetric, continuous, and coercive so that problem (2.3) is well-posed if the bilinear form $b\left( \cdot ,\cdot \right)$ satisfies the inf-sup condition for $\left( {\textbf{S}}, M \right)$.

### Definition 2.1

Let ${\textbf{S}}$ and *M* be finite-dimensional subspaces of ${\textbf{H}} _{0}^{1}\left( \Omega \right)$ and $L_{0}^{2}\left( \Omega \right)$. The pair $\left( {\textbf{S}},M\right)$ is called *inf-sup stable* if the *inf-sup constant* is positive:

2.4 $$\begin{aligned} \beta \left( {\textbf{S}},M\right) :=\inf _{q\in M\backslash \left\{ 0\right\} }\sup _{{\textbf{v}}\in {\textbf{S}}\backslash \left\{ {\textbf{0}}\right\} } \frac{\left( q,\text {div}{\textbf{v}}\right) _{L^{2}\left( \Omega \right) }}{\left\| {\textbf{v}}\right\| _{{\textbf{H}}^{1}\left( \Omega \right) }\left\| q\right\| _{L^{2}\left( \Omega \right) }}>0. \end{aligned}$$

## Pressure improved Scott–Vogelius element

Let ${\mathcal {T}}$ be a conforming, shape-regular triangulation of the domain $\Omega$ into closed triangles $K\in {\mathcal {T}}$ with diameter $h_{K}$. The set of vertices is given by ${\mathcal {V}}\left( {\mathcal {T}}\right)$ and the additional subscripts ${\mathcal {V}}_{\Omega }\left( {\mathcal {T}}\right)$, ${\mathcal {V}}_{\partial \Omega }\left( {\mathcal {T}}\right)$ specify whether a vertex is located in the domain or on its boundary. The set of edges is denoted by ${\mathcal {E}}\left( {\mathcal {T}}\right)$ and the same subscript convention as for the vertices applies. For a vertex ${\textbf{z}}\in {\mathcal {V}}\left( {\mathcal {T}}\right)$, the local vertex patch is given by

3.1 $$\begin{aligned} \begin{array}{ll} {\mathcal {T}}_{{\textbf{z}}}:=\left\{ K\in {\mathcal {T}}\mid {\textbf{z}}\in K\right\}&\text {and\quad }\omega _{{\textbf{z}}}:= {\displaystyle \bigcup \limits _{K\in {\mathcal {T}}_{{\textbf{z}}}}} K \end{array} \end{aligned}$$

with the local mesh width $h_{{\textbf{z}}}:=\max \left\{ h_{K}:K\in {\mathcal {T}}_{{\textbf{z}}}\right\}$. For any vertex ${\textbf{z}}\in {\mathcal {V}}\left( {\mathcal {T}}\right)$, we fix a local counterclockwise numbering of the $N_{{\textbf{z}}}:={\text {card}}{\mathcal {T}}_{{\textbf{z}}}$ triangles in

3.2 $$\begin{aligned} {\mathcal {T}}_{{\textbf{z}}}=\left\{ K_{j}:1\le j\le N_{{\textbf{z}}}\right\} . \end{aligned}$$

A triangle neighborhood of some triangle $K\in {\mathcal {T}}$ is given by

3.3 $$\begin{aligned} \omega \left( K\right) := {\displaystyle \bigcup \limits _{\begin{array}{c} K^{\prime }\in {\mathcal {T}}\\ K^{\prime }\cap K\ne \emptyset \end{array}}} K^{\prime }. \end{aligned}$$

The shape-regularity constant

3.4 $$\begin{aligned} \gamma _{{\mathcal {T}}}:=\max _{K\in {\mathcal {T}}}\frac{h_{K}}{\rho _{K}} \end{aligned}$$

relates the local mesh width $h_{K}$ with the diameter $\rho _{K}$ of the largest inscribed ball in an element $K\in {\mathcal {T}}$. The global mesh width is given by $h_{{\mathcal {T}}}:=\max \left\{ h_{K}:K\in {\mathcal {T}}\right\}$. For a subset $M\subset {\mathbb {R}}^{2}$, we denote the area of *M* by $\left| M\right|$. Let ${\mathbb {P}}_{k}(K)$ denote the space of polynomials on $K\in {\mathcal {T}}$ with total degree smaller than or equal to $k\in {\mathbb {N}}_{0}$ and define

3.5 $$\begin{aligned} {\mathbb {P}}_{k}\left( {\mathcal {T}}\right)&:=\left\{ q\in L^{2}\left( \Omega \right) \mid \forall K\in {\mathcal {T}}:\left. q\right| _{\overset{\circ }{{K}}}\in {\mathbb {P}}_{k}\left( \overset{\circ }{{K}}\right) \right\} , \nonumber \\ {\mathbb {P}}_{k,0}\left( {\mathcal {T}}\right)&:={\mathbb {P}}_{k}\left( {\mathcal {T}}\right) \cap L_{0}^{2}\left( \Omega \right) =\left\{ q\in {\mathbb {P}}_{k}\left( {\mathcal {T}}\right) \mid \int _{\Omega }q=0\right\} , \nonumber \\ S_{k}\left( {\mathcal {T}}\right)&:={\mathbb {P}}_{k}\left( {\mathcal {T}}\right) \cap H^{1}(\Omega ), \nonumber \\ S_{k,0}\left( {\mathcal {T}}\right)&:=S_{k}\left( {\mathcal {T}}\right) \cap H_{0}^{1}\left( \Omega \right) . \end{aligned}$$

As usual $\overset{\circ }{K}$ denotes the open interior of *K*. For any $q\in {\mathbb {P}}_{k}\left( {\mathcal {T}}\right)$, we write

3.6 $$\begin{aligned} q_{\text {mvz}}:=q-{\overline{q}}\quad \text {with the integral mean\quad }{\overline{q}}:=\frac{1}{\left| \Omega \right| }\int _{\Omega }q. \end{aligned}$$

### The Scott–Vogelius element

**Fig. 1:**
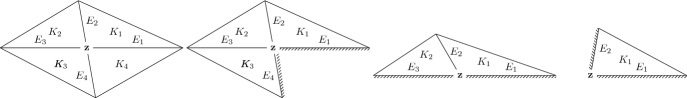
Vertex patch for an interior singular vertex ${\textbf{z}}\in {\mathcal {V}}_{\Omega }({\mathcal {T}})$ with $N_{{\textbf{z}}}=4$ (resp. boundary singular vertex ${\textbf{z}}\in {\mathcal {V}}_{\partial \Omega }({\mathcal {T}})$ with $N_{{\textbf{z}}}=1,2,3$) triangles

It is well known that the most intuitive Stokes element $\left( {\textbf{S}}_{k,0}\left( {\mathcal {T}}\right) ,{\mathbb {P}}_{k-1,0}\left( {\mathcal {T}}\right) \right)$ is in general unstable. The analysis in [21, 24] for $k\ge 4$ relates the instability of $\left( {\textbf{S}}_{k,0}\left( {\mathcal {T}}\right) ,{\mathbb {P}}_{k-1,0}\left( {\mathcal {T}}\right) \right)$ to the presence of *critical* or *singular points* in the mesh ${\mathcal {T}}$. The following definition is illustrated by Fig. 1.

#### Definition 3.1

The local measure of singularity $\Theta \left( {\textbf{z}}\right)$ at ${\textbf{z}}\in {\mathcal {V}}({\mathcal {T}})$ reads

3.7 $$\begin{aligned} \Theta \left( {\textbf{z}}\right) := {\left\{ \begin{array}{ll} \max \left\{ \left. \left| \sin \left( \theta _{i}+\theta _{i+1}\right) \right| \;\right| \;0\le i\le N_{{\textbf{z}}}\right\} &  \text {if }{\textbf{z}}\in {\mathcal {V}}_{\Omega }\left( {\mathcal {T}}\right) ,\\ \max \left\{ \left. \left| \sin \left( \theta _{i}+\theta _{i+1}\right) \right| \;\right| \;0\le i\le N_{{\textbf{z}}}-1\right\} &  \text {if }{\textbf{z}}\in {\mathcal {V}}_{\partial \Omega }({\mathcal {T}})\wedge N_{{\textbf{z}} }>1,\\ 0 &  \text {if }\mathbf {z\in }{\mathcal {V}}_{\partial \Omega }({\mathcal {T}})\wedge N_{{\textbf{z}}}=1, \end{array}\right. } \end{aligned}$$

where the angles $\theta _{j}$ in $K_{j}\in {\mathcal {T}} _{{\textbf{z}}}$ at ${\textbf{z}}$ are numbered counterclockwise from $1\le j\le N_{{\textbf{z}}}$ (see (3.2)) and cyclic numbering is applied: $\theta _{N_{{\textbf{z}}}+1}:=\theta _{1}$ if the patch is closed, i.e., ${\textbf{z}}\in {\mathcal {V}}_{\Omega }\left( {\mathcal {T}}\right)$. A vertex ${\textbf{z}}\in {\mathcal {V}}\left( {\mathcal {T}}\right)$ with $\Theta \left( {\textbf{z}}\right) =0$ is a *singular vertex* and the set of all singular vertices is

$$\begin{aligned} {\mathcal {C}}_{{\mathcal {T}}}:=\left\{ {\textbf{z}}\in {\mathcal {V}}\left( {\mathcal {T}}\right) \mid \Theta \left( {\textbf{z}}\right) =0\right\} . \end{aligned}$$

The global *measure of singularity* of the mesh ${\mathcal {T}}$ is

3.8 $$\begin{aligned} \Theta _{\min }:=\min _{{\textbf{z}}\in {\mathcal {V}}\left( {\mathcal {T}}\right) \backslash {\mathcal {C}}_{{\mathcal {T}}}}\Theta \left( {\textbf{z}}\right) . \end{aligned}$$

For any vertex ${\textbf{z}}\in {\mathcal {V}}\left( {\mathcal {T}}\right)$ and all $q\in {\mathbb {P}}_{k-1}\left( {\mathcal {T}}\right)$, the functional $A_{{\mathcal {T}},{\textbf{z}}}$ is the alternating sum

3.9 $$\begin{aligned} A_{{\mathcal {T}},{\textbf{z}}}\left( q\right) :=\sum _{\ell =1}^{N_{{\textbf{z}}} }\left( -1\right) ^{\ell }\left( \left. q\right| _{K_{\ell }}\right) \left( {\textbf{z}}\right) \end{aligned}$$

over the triangles $K_{\ell }\in {\mathcal {T}}_{{\textbf{z}}}$ for $1\le \ell \le N_{{\textbf{z}}}$.

In [21, 24], the space

3.10 $$\begin{aligned} M_{0,k-1}\left( {\mathcal {T}}\right) :=\left\{ \left. q\in {\mathbb {P}} _{k-1,0}\left( {\mathcal {T}}\right) \;\right| \;\forall {\textbf{z}} \in {\mathcal {C}}_{{\mathcal {T}}}:\ A_{{\mathcal {T}},{\textbf{z}}}\left( q\right) =0\right\} \end{aligned}$$

was introduced and used in the definition of the Scott–Vogelius element $\left( {\textbf{S}}_{k,0}\left( {\mathcal {T}}\right) ,M_{0,k-1}\left( {\mathcal {T}}\right) \right)$. It was proven in [24] that this element enjoys two important properties of a “good” Stokes element: a) inf-sup stability which follows from

3.11 $$\begin{aligned} {\text {div}}{\textbf{S}}_{k,0}\left( {\mathcal {T}}\right) =M_{0,k-1} \left( {\mathcal {T}}\right) \end{aligned}$$

and b) the discrete velocity is divergence free. A third important property certainly is the approximation property of the discrete spaces. Since ${\textbf{S}}_{k,0}\left( {\mathcal {T}}\right)$ is a standard finite element space, its approximation property is well known. For the pressure space, however, the approximation property might deteriorate in the vicinity of vertices having a particular type of vertex patch ${\mathcal {T}}_{{\textbf{z}}}$ as explained next. We start with an observation for $q\in M_{0,k-1}\left( {\mathcal {T}}\right)$. Suppose ${\textbf{z}}\in {\mathcal {C}}_{{\mathcal {T}}} \cap \partial \Omega$ is a singular boundary vertex with an odd number of neighboring triangles (type 2 or 4 in Fig. 1). The side condition in the definition of the pressure space $A_{{\mathcal {T}},{\textbf{z}}}\left( q\right) =0$ reveals the following implication

3.12 $$\begin{aligned} q\in M_{0,k-1}\left( {\mathcal {T}}\right) \text { is continuous at } {\textbf{z}}\implies q\left( {\textbf{z}}\right) =0. \end{aligned}$$

Since the exact pressure does not vanish at theses points in general, we cannot expect a good approximation property of $M_{0,k-1}\left( {\mathcal {T}}\right)$ in neighborhoods of such vertices. In particular, the $L^{\infty }$ norm $\left\| p-p_{M}\right\| _{L^{\infty }\left( \Omega \right) }$ of a smooth pressure $p\in C^{0}\left( \overline{\Omega }\right)$ is at least $\left| p\left( {\textbf{z}}\right) \right|$, independent of the triangulation ${\mathcal {T}}$. This is a drawback and we will present a strategy to modify the pressure space in these vertices such that standard approximation properties hold. The singular vertices responsible for the deficiency in the approximation properties of $M_{0,k-1}\left( {\mathcal {T}}\right)$ are collected in the set of *super-critical* vertices:

3.13 $$\begin{aligned} \mathcal{S}\mathcal{C}_{{\mathcal {T}}}:=\left\{ \left. {\textbf{z}}\in {\mathcal {C}} _{{\mathcal {T}}}\;\right| \;N_{{\textbf{z}}}\text { is odd}\right\} . \end{aligned}$$

Since $N_{{\textbf{z}}} \in \left\{ 1,3\right\}$, super-critical vertices always lie on the boundary (cf., Fig. 1).

#### Remark 3.2

It was shown in [1] that the space

3.14 $$\begin{aligned} {\widetilde{Q}}_{0}^{h,k}:=\left\{ \left. q\in {\mathbb {P}}_{k-1,0}\left( {\mathcal {T}}\right) \;\right| \;q\text { is }C^{0}\text { at all vertices}\right\} \end{aligned}$$

has optimal approximation properties. Given any $q\in {\widetilde{Q}}_{0}^{h,k}$, Definition 3.1 provides $A_{{\mathcal {T}},{\textbf{z}}}\left( q\right) =0$ for all ${\textbf{z}}\in {\mathcal {C}}_{{\mathcal {T}}}$ with $N_{{\textbf{z}}}$ even. Therefore, we only expect the loss of approximability in the vertices in ${{\mathcal {S}}}{{\mathcal {C}}}_{{\mathcal {T}}}$.

As discussed in the introduction, we will present a simple modification strategy to remedy the reduced approximation properties. For the Scott–Vogelius element, this strategy leads to a local postprocessing step and recovers the optimal convergence order for the pressure approximation.

### A simple postprocessing strategy

Since the discrete pressure space $M_{0,k-1}\left( {\mathcal {T}}\right)$ is defined by restricting the full pressure space ${\mathbb {P}}_{k-1,0}\left( {\mathcal {T}}\right)$ at singular vertices via the functional $A_{{\mathcal {T}},{\textbf{z}}}$, the complement of $M_{0,k-1}\left( {\mathcal {T}}\right)$ in ${\mathbb {P}}_{k-1,0}\left( {\mathcal {T}}\right)$ is non-trivial in the presence of singular vertices and has been described in [4] and [9, Def. 3.11].

#### Definition 3.3

For a vertex ${\textbf{z}}\in {\mathcal {V}}\left( {\mathcal {T}} \right)$, the *critical function *$b_{k-1,{\textbf{z}}}\in {\mathbb {P}} _{k-1}\left( {\mathcal {T}}\right)$ is given by

3.15 $$\begin{aligned} b_{k-1,{\textbf{z}}}=\sum _{\ell =1}^{N_{{\textbf{z}}}}\frac{\left( -1\right) ^{k-1+\ell }}{\left| K_{\ell }\right| }P_{k-1}^{\left( 0,2\right) }\left( 1-2\lambda _{K_{\ell },{\textbf{z}}}\right) \chi _{K_{\ell } }, \end{aligned}$$

where $\chi _{K_{\ell }}$ is the characteristic function of the triangle $K_{\ell }\in {\mathcal {T}}_{{\textbf{z}}}$ and $P_{k-1}^{\left( \alpha ,\beta \right) }$ is the Jacobi polynomial used here for the parameters $\alpha =0$, $\beta =2$ (cf., [17, Table 18.3.1]). The barycentric coordinate for a triangle $K\in {\mathcal {T}}\left( {\textbf{z}}\right)$ corresponding to the vertex ${\textbf{z}}$ is denoted by $\lambda _{K,{\textbf{z}} }$.

The following properties of the critical functions $b_{k-1,{\textbf{z}}}$ were proven in [13, Lem. 6].

#### Proposition 3.4

The critical functions $b_{k-1,{\textbf{z}}}$ satisfy for all $K_{\ell }\in {\mathcal {T}}_{{\textbf{z}}}:$

3.16a $$\begin{aligned} \left( b_{k-1,{\textbf{z}}},1\right) _{L^{2}\left( K_{\ell }\right) }&=\left( -1\right) ^{\ell }\left( {\begin{array}{c}k+1\\ 2\end{array}}\right) ^{-1}, \end{aligned}$$

3.16b $$\begin{aligned} \left\| b_{k-1,{\textbf{z}}}\right\| _{L^{2}\left( K_{\ell }\right) }^{2}&=\left| K_{\ell }\right| ^{-1}, \end{aligned}$$

3.16c $$\begin{aligned} \left. b_{k-1,{\textbf{z}}}\right| _{K_{\ell }}\left( {\textbf{y}}\right)&=\frac{\left( -1\right) ^{\ell }}{\left| K_{\ell }\right| }\left\{ \begin{array}{ll} \left( {\begin{array}{c}k+1\\ 2\end{array}}\right) &  \text {if }{\textbf{y}}={\textbf{z}},\\ \left( -1\right) ^{k-1} & \text {if } {\textbf{y}} \in {\mathcal {V}} \left( {\mathcal {T}}_{{\textbf{z}}} \right) \setminus \left\{ {\textbf{z}} \right\} , \end{array} \right. \end{aligned}$$

3.16d $$\begin{aligned} A_{{\mathcal {T}},{\textbf{z}}}\left( b_{k-1,{\textbf{z}}}\right)&=\left( {\begin{array}{c}k+1\\ 2\end{array}}\right) \left\| b_{k-1,{\textbf{z}}}\right\| _{L^{2}\left( \omega _{{\textbf{z}}}\right) }^{2}. \end{aligned}$$

For $k\ge 2$, the set $\left\{ b_{k-1,{\textbf{z}}}:{\textbf{z}}\in {\mathcal {V}}\left( {\mathcal {T}}\right) \right\} \cup \left\{ 1\right\}$ is linearly independent and satisfies for any $c_{\textbf{z}}\in \mathbb R, \textbf{z}\in \mathcal V(K)$, and $K\in \mathcal T$ the estimate

3.17 $$\begin{aligned} \frac{3}{4}\left\| \sum _{{\textbf{z}}\in {\mathcal {V}}\left( K\right) }c_{{\textbf{z}}}b_{k,{\textbf{z}}}\right\| _{L^{2}\left( K\right) }^{2} \le \left| K\right| ^{-1}\sum _{{\textbf{z}}\in {\mathcal {V}}\left( K\right) }c_{{\textbf{z}}}^{2}\le \frac{12}{7}\min _{\alpha \in {\mathbb {R}} }\left\| \sum _{{\textbf{z}}\in {\mathcal {V}}\left( K\right) }c_{{\textbf{z}} }b_{k,{\textbf{z}}}-\alpha \right\| _{L^{2}\left( K\right) }^{2}. \nonumber \\ \end{aligned}$$

The functions $b_{k-1,{\textbf{z}}}$ characterise the orthogonal complement of $M_{0,k-1}\left( {\mathcal {T}}\right)$ in ${\mathbb {P}}_{k-1,0}\left( {\mathcal {T}}\right)$. Let $\oplus$ denote the direct sum of vector spaces and recall $\left( \cdot \right) _{{\text {mvz}}}$ from (3.6).

#### Proposition 3.5

([9, Lem. 3.13]) The decomposition

$$\begin{aligned} {\mathbb {P}}_{k-1,0}\left( {\mathcal {T}}\right) =M_{0,k-1}\left( {\mathcal {T}} \right) \oplus {\text {span}}\left\{ \left( b_{k-1,{\textbf{z}}}\right) _{{\text {mvz}}}\mid {\textbf{z}}\in {\mathcal {C}}_{{\mathcal {T}}}\right\} \end{aligned}$$

is $L^2$ orthogonal, i.e., any $q_M\in M_{0,k-1}({\mathcal {T}})$ satisfies $(q_M, \left( b_{k-1,{\textbf{z}}}\right) _{{\text {mvz}}})_{L^2(\Omega )}=0$ for all ${\textbf{z}}\in {\mathcal {C}}_{{\mathcal {T}}}$.

#### Proof

This is a direct consequence of [9, Lem. 3.13], the definition of $\left( \cdot \right) _{{\text {mvz}}}$, and the integral mean zero condition in $M_{0,k-1}({\mathcal {T}})$; further details are omitted. $\square$

We will employ a continuous, linear functional

3.18 $$\begin{aligned} f_{{\textbf{z}}}:{\mathbb {P}}_{k-1}\left( {\mathcal {T}}\right) \rightarrow \mathbb {R\quad }\text {with }\left| f_{{\textbf{z}}}\left( q\right) \right| \le \frac{C_{f_{{\textbf{z}}}}}{\left\| b_{k-1,{\textbf{z}} }\right\| _{L^{2}\left( \Omega \right) }}\left\| q\right\| _{L^{2}\left( \Omega \right) } \end{aligned}$$

to define the modified pressure space $M_{0,k-1}^{{\text {mod}}}\left( {\mathcal {T}}\right) \subset {\mathbb {P}}_{k-1,0}\left( {\mathcal {T}}\right)$ by:

3.19 $$\begin{aligned} M_{0,k-1}^{{\text {mod}}}\left( {\mathcal {T}}\right) :=\left\{ q+\sum _{{\textbf{z}}\in {{\mathcal {S}}}{{\mathcal {C}}}_{{\mathcal {T}}}}f_{{\textbf{z}}}\left( q\right) \left( b_{k-1,{\textbf{z}}}\right) _{{\text {mvz}}}:\;q\in M_{0,k-1}\left( {\mathcal {T}}\right) \right\} . \end{aligned}$$

The modification in $M_{0,k-1}^{{\text {mod}}}\left( {\mathcal {T}}\right)$ for general $f_{{\textbf{z}}}$ overcomes the implication (3.12) that results in the suboptimal approximation properties of $M_{0,k-1}\left( {\mathcal {T}}\right)$ in the presence of super-critical vertices and defines a novel discretisation of (2.2). A *good* choice of $f_{{\textbf{z}}}$ discussed in Sect. 3.3 even enables optimal approximation properties of $M_{0,k-1}^{{\text {mod}}}\left( {\mathcal {T}}\right)$ in the sense of Theorem 3.14 below.

#### Definition 3.6

Given $k\in {\mathbb {N}}$ and functionals $f_{{\textbf{z}}}$ with (3.18) for all ${\textbf{z}}\in {\mathcal {C}}_{{\mathcal {T}}}$ and $M_{0,k-1}^{{\text {mod}}}({\mathcal {T}})$ from (3.19), *the pressure-improved Scott–Vogelius element* is given by the pair $\left( {\textbf{S}}_{k,0}\left( {\mathcal {T}}\right) ,M_{0,k-1}^{{\text {mod}} }\left( {\mathcal {T}}\right) \right)$.

An important point is that the pressure-improved Scott–Vogelius solution is a simple post-processing of the classical Scott–Vogelius solution with possibly better approximation properties.

#### Theorem 3.7

Let $k\ge 4$ and ${\textbf{F}}\in {\textbf{H}}^{-1}\left( \Omega \right)$. Then $\left( {\textbf{u}}_{{\textbf{S}}},p_{M}\right) \in {\textbf{S}}_{k,0}\left( {\mathcal {T}}\right) \times M_{0,k-1}\left( {\mathcal {T}}\right)$ solves (2.3) in $({\textbf{S}}_{k,0}\left( {\mathcal {T}}\right) ,M_{0,k-1}\left( {\mathcal {T}}\right) )$ if and only if $\left( {\textbf{u}}_{{\textbf{S}}},p_{M}^*\right)$ solves (2.3) in $({\textbf{S}}_{k,0}\left( {\mathcal {T}}\right) ,M_{0,k-1}^{{\text {mod}}}\left( {\mathcal {T}}\right) )$ with

3.20 $$\begin{aligned} p_{M}^{*}:=p_{M}+\sum _{{\textbf{z}}\in {{\mathcal {S}}}{{\mathcal {C}}}_{{\mathcal {T}}} }f_{{\textbf{z}}}\left( p_{M}\right) \left( b_{k-1,{\textbf{z}}}\right) _{{\text {mvz}}}\in M_{0,k-1}^{{\text {mod}}}\left( {\mathcal {T}}\right) . \end{aligned}$$

For $C_f{:}{=}1+\sum  _{{\textbf{z}}\in {\mathcal {S}}{\mathcal {C}}_{{\mathcal {T}}}} C_{f_{{\textbf{z}}}}$, the modified discrete pressure $p_{M}^{*}$ satisfies the error estimate

3.21 $$\begin{aligned} \left\| p-p_{M}^{*}\right\| _{L^{2}\left( \Omega \right) }\le&\frac{C_{{\text {vel}}}C_f^{2}}{\Theta _{\min }^{2}} \inf _{{\textbf{v}}\in {\textbf{S}}_{k,0}\left( {\mathcal {T}}\right) }\left\| {\textbf{u}}-{\textbf{v}}\right\| _{H^{1}\left( \Omega \right) } +\frac{C_{{\text {pres}}}C_f}{\Theta _{\min }}\inf _{q\in M_{0,k-1} ^{{\text {mod}}}\left( {\mathcal {T}}\right) }\left\| p-q\right\| _{L^{2}\left( \Omega \right) } \end{aligned}$$

with the continuous solution $\left( {\textbf{u}},p\right) \in {\textbf{H}}_{0}^{1}\left( \Omega \right) \times L_{0}^{2}\left( \Omega \right)$ to (2.2). The positive constants $C_{{\text {vel}}},C_{{\text {pres}}}$ only depend on the shape-regularity of the mesh and the domain $\Omega$.

The proof of Theorem 3.7 below is preceded by the well-posedness of the discrete problem (2.3) for the pressure-improved Scott–Vogelius element that is a consequence of the inf-sup stability inherited from the classical Scott–Vogelius element. The following proposition recalls a right-inverse of the divergence from [2] that is bounded in terms of $\Theta _{\min }^{-1}$ with $\Theta _{\min }$ from (3.8). The construction of a right-inverse goes back to [21, 24] while in [15] a definition is presented which allows for optimal bounds with respect to *h*, *k* and $\Theta _{\min }$.

#### Proposition 3.8

([2]) For $k\ge 4$, there is a linear operator $\Pi _{k}:M_{0,k-1}\left( {\mathcal {T}}\right) \rightarrow {\textbf{S}}_{k,0}\left( {\mathcal {T}}\right)$ with

3.22 $$\begin{aligned} {\text {div}}\Pi _{k}q=q\quad \text{ and }\quad \left\| \Pi _{k}q\right\| _{{{\textbf {H}}}^{1}\left( \Omega \right) }\le \frac{\left\| q\right\| _{L^{2}\left( \Omega \right) }}{ c\,\Theta _{\min }}\quad \forall q\in M_{0,k-1}\left( {\mathcal {T}}\right) . \end{aligned}$$

The constant *c* only depends on $\Omega$ and the shape-regularity of the mesh. The inf-sup constant for the Scott–Vogelius element $\left( {\textbf{S}}_{k,0}\left( {\mathcal {T}}\right) ,M_{0,k-1}\left( {\mathcal {T}} \right) \right)$ is bounded from below by $c\Theta _{\min }$.

Recall the inf-sup constant $\beta$ from (2.4), $C_f$ from Theorem 3.7, and *c* from Proposition 3.8.

#### Lemma 3.9

Let $k\ge 4$. The pressure-improved Scott–Vogelius element is inf-sup stable with

3.23 $$\begin{aligned} \beta \big ( {\textbf{S}}_{k,0}\left( {\mathcal {T}}\right) ,M_{0,k-1}^{{\text {mod}}}\left( {\mathcal {T}}\right) \big ) \ge c\Theta _{\min }/C_f. \end{aligned}$$

#### Proof

The operator ${\mathcal {E}}_{k}:M_{0,k-1}\left( {\mathcal {T}} \right) \rightarrow M_{0,k-1}^{{\text {mod}}}\left( {\mathcal {T}}\right)$ given for any $q\in M_{0,k-1}({\mathcal {T}})$ by

3.24 $$\begin{aligned} {\mathcal {E}}_{k}q=q+\sum _{{\textbf{z}}\in {{\mathcal {S}}}{{\mathcal {C}}}_{{\mathcal {T}}}} f_{{\textbf{z}}}\left( q\right) \left( b_{k-1,{\textbf{z}}}\right) _{{\text {mvz}}} \end{aligned}$$

is surjective onto $M_{0,k-1}^{{\text {mod}}}\left( {\mathcal {T}}\right)$ by (3.19). Proposition 3.5 reveals for any $q\in M_{0,k-1}({\mathcal {T}})$ that

3.25 $$\begin{aligned} \left( {\mathcal {E}}_{k}q,q\right) _{L^{2}\left( \Omega \right) }&=\Vert q\Vert _{L^{2}\left( \Omega \right) }^2+\sum _{{\textbf{z}} \in {{\mathcal {S}}}{{\mathcal {C}}}_{{\mathcal {T}}}}f_{{\textbf{z}}}\left( q\right) \left( \left( b_{k-1,{\textbf{z}}}\right) _{{\text {mvz}}},q\right) _{L^{2}\left( \Omega \right) } =\Vert q\Vert _{L^{2}\left( \Omega \right) }^2. \end{aligned}$$

Triangle inequalities, the boundedness of $f_{{\textbf{z}}}$ from (3.18), and $C_f=1+\sum  _{{\textbf{z}}\in {\mathcal {S}}{\mathcal {C}}_{{\mathcal {T}}}} C_{f_{{\textbf{z}}}}$ show

$$\begin{aligned} \left\| {\mathcal {E}}_{k}q\right\| _{L^{2}\left( \Omega \right) } \le \left\| q\right\| _{L^{2}\left( \Omega \right) }+\sum _{{\textbf{z}}\in {{\mathcal {S}}}{{\mathcal {C}}}_{{\mathcal {T}}}}\left| f_{{\textbf{z}}}\left( q\right) \right| \left\| b_{k-1,{\textbf{z}}}\right\| _{L^{2}\left( \omega _{{\textbf{z}}}\right) }\le C_f \left\| q\right\| _{L^{2}\left( \Omega \right) }. \end{aligned}$$

This, the surjectivity of ${\mathcal {E}}_k$, and the choice ${\textbf{v}} _{q}:=\Pi _{k}q\in {\textbf{S}}_{k,0}({\mathcal {T}})$ with ${\text {div}}({\textbf{v}}_q)=q$ and $c\Theta _{\min }\Vert {\textbf{v}}_q\Vert _{{\textbf{H}}^1(\Omega )}\le \Vert q\Vert _{L^2(\Omega )}$ from Proposition 3.8 for any $q\in M_{0,k-1}({\mathcal {T}})$ verify

$$\begin{aligned} \inf _{q^{*}\in M_{0,k-1}^{{\text {mod}}}\left( {\mathcal {T}}\right) \setminus \left\{ 0\right\} }\sup _{{\textbf{v}}\in {\textbf{S}}_{k,0}\left( {\mathcal {T}}\right) \setminus \left\{ {\textbf{0}}\right\} }\frac{\left( q^{*},{\text {div}}{\textbf{v}}\right) _{L^{2}\left( \Omega \right) } }{\left\| q^{*}\right\| _{L^{2}\left( \Omega \right) }\left\| {\textbf{v}}\right\| _{H^{1}\left( \Omega \right) }}&\ge \inf _{q\in M_{0,k-1}\left( {\mathcal {T}}\right) \setminus \left\{ 0\right\} } \frac{\left( {\mathcal {E}}_{k}q,q\right) _{L^{2}\left( \Omega \right) }}{\left\| {\mathcal {E}}_{k}q\right\| _{L^{2}\left( \Omega \right) }\left\| {\textbf{v}}_{q}\right\| _{H^{1}\left( \Omega \right) }}\\&\ge c\Theta _{\min }/C_f. \qquad \qquad \qquad \qquad \qquad \qquad \qquad \qquad \square \end{aligned}$$

#### Proof of Theorem 3.7

Given any ${\textbf{v}}\in {\textbf{S}}_{k,0}\left( {\mathcal {T}}\right)$, Proposition 3.5 verifies as in (3.25) that

$$\begin{aligned} b\left( {\textbf{v}},p^{*}_{M}\right) =b\left( {\textbf{v}},p_M\right) +\sum _{{\textbf{z}}\in {{\mathcal {S}}}{{\mathcal {C}}}_{{\mathcal {T}}}}f_{{\textbf{z}}}\left( p_M\right) b\left( {\textbf{v}},\left( b_{k-1,{\textbf{z}}}\right) _{{\text {mvz}}}\right) =b\left( {\textbf{v}},p_M\right) . \end{aligned}$$

Since $({\textbf{u}}_{{\textbf{S}}},p_M)$ solves (2.3) for ${\textbf{S}}={\textbf{S}}_{k,0}({\mathcal {T}})$ and $M=M_{0,k-1}({\mathcal {T}})$ with ${\text {div}} {\textbf{u}}_S = 0$, this verifies that $\left( {\textbf{u}}_{{\textbf{S}}}, p_M^{*} \right)$ solves (2.3) for ${\textbf{S}}={\textbf{S}}_{k,0}({\mathcal {T}})$ and $M=M_{0,k-1}^{{\text {mod}}}({\mathcal {T}})$. The inf-sup stability from Proposition 3.8 and Lemma 3.9 verify the uniqueness of the respective discrete solutions. The pressure estimate follows from [6, Chap. 5, Thm. 5.2.3, (5.2.27)] in combination with (3.23). $\square$

The *hp*-explicit estimates of the first infimum $\inf _{{\textbf{v}} \in {\textbf{S}}_{k,0}\left( {\mathcal {T}}\right) }\left\| {\textbf{u}} -{\textbf{v}}\right\| _{H^{1}\left( \Omega \right) }$ in (3.21) for functions ${\textbf{u}}$ with certain Sobolev smoothness are well known from the literature on *hp* finite elements. Here, our focus will be on the estimate of the second infimum in (3.21) related to our new pressure space.

### Assumptions for and examples of functionals $f_{{\textbf{z}}}$

**Fig. 2:**
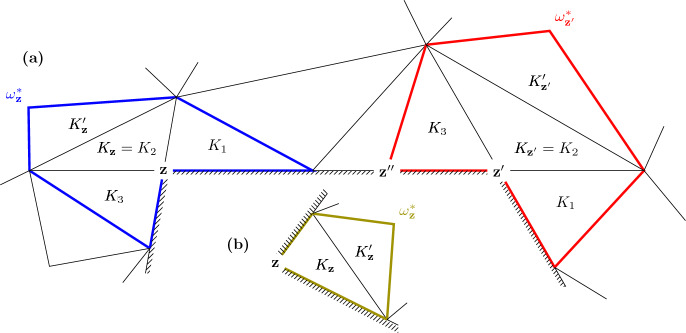
(**a**) The extended vertex patch $\omega _{{\textbf{z}}}^{*}$ (contoured by blue lines) is of Robinson type, whereas the extended vertex patch $\omega _{{\textbf{z}}^{\prime }}^{*}$ (outlined in red) is not of Robinson type due to presence of ${\textbf{z}}^{\prime \prime } \in {\mathcal {C}}_{{\mathcal {T}}} {\setminus } {{\mathcal {S}}}{{\mathcal {C}}}_{{\mathcal {T}}}$. Observe that ${\textbf{z}}, {\textbf{z}}^{\prime } \in {{\mathcal {S}}}{{\mathcal {C}}}_{{\mathcal {T}}}$ satisfy (i) in Definition 3.11. (**b**) The extended vertex patch $\omega _{{\textbf{z}}}^{*}$ is of Robinson type for ${\textbf{z}} \in {{\mathcal {S}}}{{\mathcal {C}}}_{{\mathcal {T}}}$ with $N_{{\textbf{z}}} = 1$ (contoured in olive) (Color figure online)

In order to derive approximation properties for the modified discrete pressure space $M_{0,k-1}^{{\text {mod}}}\left( {\mathcal {T}}\right)$, we will specify the functionals $f_{{\textbf{z}}}$ in a concrete way. In order to reduce technicalities, we impose some simplifying assumptions on the triangulation (see, Fig. 2). Take any ${\textbf{z}}\in {{\mathcal {S}}}{{\mathcal {C}}}_{{\mathcal {T}}}$ with nodal patch ${\mathcal {T}}_{{\textbf{z}}}:=\left\{ K_{j}:1\le j\le N_{{\textbf{z}}}\right\}$ and set $K_{{\textbf{z}}}\in {\mathcal {T}}_{{\textbf{z}}}$ to be

3.26 $$\begin{aligned} K_{{\textbf{z}}}:= {\left\{ \begin{array}{ll} K_{1} &  \text {if }N_{{\textbf{z}}}=1,\\ K_{2} &  \text {if }N_{{\textbf{z}}}=3. \end{array}\right. } \end{aligned}$$

#### Assumption 3.10

For any ${\textbf{z}}\in {{\mathcal {S}}}{{\mathcal {C}}}_{{\mathcal {T}}}$ with $K_{{\textbf{z}}}$ as in (3.26), there exists a triangle denoted by $K_{{\textbf{z}}}^{\prime }\in {\mathcal {T}}$ which is adjacent to $K_{{\textbf{z}}}$ but not contained in ${\mathcal {T}}_{{\textbf{z}}}$; see Fig. 2 for reference.

Assumption 3.10 allows us to define the extended nodal patch for ${\textbf{z}}\in {{\mathcal {S}}}{{\mathcal {C}}}_{{\mathcal {T}}}$ by

$$\begin{aligned} {\mathcal {T}}_{{\textbf{z}}}^{*}:={\mathcal {T}}_{{\textbf{z}}}\cup \left\{ K_{{\textbf{z}}}^{\prime }\right\} \quad \text {and\quad }\omega _{{\textbf{z}}} ^{*}:=\omega _{{\textbf{z}}}\cup K_{{\textbf{z}}}^{\prime }. \end{aligned}$$

To reduce technicalities, we restrict the discussion to meshes where super-critical vertices are properly separated. The definition is illustrated by Fig. 2.

#### Definition 3.11

Let Assumption 3.10 hold. An *isolated* super-critical vertex ${\textbf{z}} \in {{\mathcal {S}}}{{\mathcal {C}}}_{{\mathcal {T}}}$ is characterized by the conditions:

1. ${\mathcal {T}}_{{\textbf{z}}}^{*}\cap {\mathcal {T}}_{{\textbf{y}}}^{*}=\emptyset$ for all ${\textbf{y}}\in {{\mathcal {S}}}{{\mathcal {C}}}_{{\mathcal {T}}}{\setminus }\left\{ {\textbf{z}}\right\}$ and
2. $$\omega _{{\textbf{z}}}^{*}\cap {\mathcal {C}}_{{\mathcal {T}}}=\left\{ {\textbf{z}}\right\} $$

An isolated, super-critical vertex ${\textbf{z}} \in {{\mathcal {S}}}{{\mathcal {C}}}_{{\mathcal {T}}}$ is called a *Robinson vertex* (or of *Robinson type*).

#### Remark 3.12

We assume below that all super critical vertices are of Robinson type to reduce technicalities: If ${\textbf{z}}\in {\mathcal {S}}{\mathcal {C}}_{{\mathcal {T}}}$ is a Robinson vertex, the critical function $b_{k-1,{\textbf{z}}}$ satisfies

(i) $(b_{k-1,{\textbf{z}}}, b_{k-1,{\textbf{y}}})_{L^2(\Omega )}=0$ for all ${\textbf{y}}\in {\mathcal {C}}_{{\mathcal {T}}}\setminus \{{\textbf{z}}\}$ and
(ii) $b_{k-1,{\textbf{z}}}|_{K_{\textbf{y}}'}=0$ for all ${\textbf{y}}\in {\mathcal {S}}{\mathcal {C}}_{{\mathcal {T}}}$.

These properties allow us to investigate the approximation property locally in a vicinity of Robinson vertices and avoid clusters of super critical vertices with additional coupling effects.

The following *structural* assumption on $f_{{\textbf{z}}}:{\mathbb {P}}_{k-1}({\mathcal {T}})\rightarrow {\mathbb {R}}$ from (3.18) leaves considerable freedom for the particular choice and enables optimal approximation properties of $M_{0,k-1}^{{\text {mod}}}({\mathcal {T}})$ in Theorem 3.14 below. For a polynomial $q\in {\mathbb {P}}_{k}\left( K\right)$ we denote its analytic extension to ${\mathbb {R}}^{2}$ by $q^{{\text {ext}}}$.

#### Assumption 3.13

Let with Assumption 3.10 hold and $f_{{\textbf{z}}}:{\mathbb {P}}_{k-1}({\mathcal {T}})\rightarrow {\mathbb {R}}$ be given in terms of continuous linear functionals $J_{{\textbf{z}}}:{\mathbb {P}}_{k-1}(\mathbb {R}^2)\rightarrow {\mathbb {R}}$ by

3.27 $$\begin{aligned} f_{{\textbf{z}}}\left( q\right) :=J_{{\textbf{z}}}\left( \left. q\right| _{K_{{\textbf{z}}}^{\prime }}^{{\text {ext}}}\right) -J_{{\textbf{z}}}\left( \left. q\right| _{K_{{\textbf{z}}}}^{{\text {ext}}}\right) \end{aligned}$$

for any ${\textbf{z}}\in {\mathcal {S}}{\mathcal {C}}_{{\mathcal {T}}}$. Each of the functionals $J_{\textbf{z}}$ satisfies

3.28 $$\begin{aligned} J_{{\textbf{z}}}\left( \left. b_{k-1,{\textbf{z}}}\right| _{K_{{\textbf{z}}} }^{{\text {ext}}}\right) =1\quad \text {and}\quad \left| J_{{\textbf{z}}}\left( q\right) \right| \le \frac{C_{{\textbf{z}}}}{\left\| b_{k-1,{\textbf{z}}}\right\| _{L^{2}\left( \omega _{{\textbf{z}}}\right) }}\left\| q\right\| _{L^{2}\left( {\mathcal {U}}_{{\textbf{z}}}\right) }\quad \forall q\in {\mathbb {P}}_{k-1}\left( {\mathbb {R}}^{2}\right) \end{aligned}$$

for some constant $C_{{\textbf{z}}}$ which is independent of *q* and $h_{{\textbf{z}}}$ but, possibly, depends on *k*, $\Omega$, and $\gamma _{{\mathcal {T}}}$ and some subset ${\mathcal {U}}_{{\textbf{z}}}\subset K_{{\textbf{z}}}\cup K_{{\textbf{z}}}^{\prime }$ with the following property. There exists some $\delta _{{\textbf{z}}}\ge 0$ and triangles $K_{{\textbf{z}}} ^{{\text {ext}}}$, $K_{{\textbf{z}}}^{\prime ,{\text {ext}}} \subseteq {\mathbb {R}}^{2}$ not necessarily contained in ${\mathcal {T}}$ or $\Omega$ such that

3.29a $$\begin{aligned} K_{{\textbf{z}}}\cup {\mathcal {U}}_{{\textbf{z}}}&\subset K_{{\textbf{z}} }^{{\text {ext}}}\subset \left\{ {\textbf{y}}\in {\mathbb {R}}^{2} \mid {\text {dist}}\left( {\textbf{y}},K_{{\textbf{z}}}\right) \le \delta _{{\textbf{z}}}h_{K_{{\textbf{z}}}}\right\} , \end{aligned}$$

3.29b $$\begin{aligned} K_{{\textbf{z}}}^{\prime }\cup {\mathcal {U}}_{{\textbf{z}}}&\subset K_{{\textbf{z}} }^{\prime ,{\text {ext}}}\subset \left\{ {\textbf{y}}\in {\mathbb {R}}^{2} \mid {\text {dist}}\left( {\textbf{y}},K_{{\textbf{z}}}^{\prime }\right) \le \delta _{{\textbf{z}}}h_{K_{{\textbf{z}}}^{\prime }}\right\} . \end{aligned}$$

**Fig. 3:**
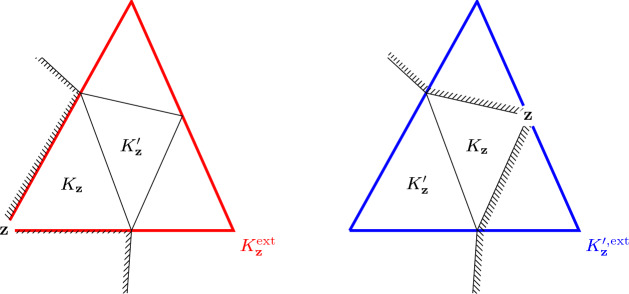
Ilustration of the extended triangles $K_{{\textbf{z}}}^{{\text {ext}}}$ (left in red) and $K_{{\textbf{z}}}^{\prime ,{\text {ext}}}$ (right in blue) for the case ${\textbf{z}} \in {{\mathcal {S}}}{{\mathcal {C}}}_{{\mathcal {T}}}$ with $N_{{{\textbf {z}}}} = 1.$ (Color figure online)

This is illustrated in Fig. 3.

Next we introduce an overlap constant for some local neighborhoods of super-critical vertices. For ${\textbf{z}}\in {{\mathcal {S}}}{{\mathcal {C}}}_{{\mathcal {T}}}$ define the regions (cf. (3.3))

3.30 $$\begin{aligned} {\widetilde{\omega }}_{{\textbf{z}}}:=\omega \left( K_{{\textbf{z}}}\right) \cup \omega \left( K_{{\textbf{z}}}^{\prime }\right) \cup K_{{\textbf{z}} }^{{\text {ext}}}\cup K_{{\textbf{z}}}^{\prime ,{\text {ext}}}. \end{aligned}$$

The maximal overlap is described by the constant

3.31 $$\begin{aligned} \max _{{\textbf{z}}\in {{\mathcal {S}}}{{\mathcal {C}}}_{{\mathcal {T}}}}{\text {card}}\left\{ {\textbf{y}}\in {{\mathcal {S}}}{{\mathcal {C}}}_{{\mathcal {T}}}\mid \left| \widetilde{\omega }_{{\textbf{z}}}\cap {\widetilde{\omega }}_{{\textbf{y}}}\right| >0\right\} =:C_{{\text {ov}}}. \end{aligned}$$

Note that it is a very mild assumption on the mesh to assume that $C_{{\text {ov}}}$ is moderately bounded; all constants in the sequel may depend on $C_{{\text {ov}}}$. Note that the constants

3.32 $$\begin{aligned} C_{J}:=\max _{{\textbf{z}}\in {{\mathcal {S}}}{{\mathcal {C}}}_{{\mathcal {T}}}}C_{{\textbf{z}}} \quad \text {and\quad }\delta _{\max }:=\max _{{\textbf{z}}\in {{\mathcal {S}}}{{\mathcal {C}}} _{{\mathcal {T}}}}\delta _{{\textbf{z}}} \end{aligned}$$

are independent of ${\textbf{z}}\in {{\mathcal {S}}}{{\mathcal {C}}}_{{\mathcal {T}}}$ and $h_{{\textbf{z}}}$ but, possibly, depend on *k*, $\Omega$ and $\gamma _{{\mathcal {T}}}$ (see Assumption 3.13).

### The approximation property for the improved pressure space $M_{0,k-1}^{{\text {mod}}}\left( {\mathcal {T}}\right)$

This subsection verifies the optimal approximation properties of the modified pressure space $M_{0,k-1}^{{\text {mod}}} \left( {\mathcal {T}} \right)$ from (3.19) under Assumption 3.13 with explicit constants in terms of the mesh width. Consider the continuous Stein extension ${\mathcal {E}}_{{\text {Stein}}}:C^{\infty }\left( {\overline{\Omega }}\right) \rightarrow C^{\infty }\left( {\mathbb {R}}^{2}\right)$ from [22, Thm. 5, p. 181] that extends, for any $m\ge 0$, to a continuous operator ${\mathcal {E}}_{{\text {Stein}}}:H^{m}\left( \Omega \right) \rightarrow H^{m}\left( {\mathbb {R}}^{2}\right)$ with

3.33 $$\begin{aligned} \left\| {\mathcal {E}}_{{\text {Stein}}}u\right\| _{H^{m}\left( {\mathbb {R}}^{2}\right) }\le C_{{\text {Stein}}}\left\| u\right\| _{H^{m}\left( \Omega \right) }\quad \forall u\in H^{m}\left( \Omega \right) . \end{aligned}$$

The constant $C_{{\text {Stein}} }$ depends on *m* and $\Omega$. Let $T_{k}\in {\mathbb {P}}_k({\mathbb {R}})$ denote the Chebyshev polynomial of first kind and degree *k*; see [17, Table 18.3.1] for details.

#### Theorem 3.14

Let Assumption 3.13 hold and suppose that all super-critical vertices are of Robinson type. For any $p\in H^{s-1}\left( \Omega \right) \cap L_{0}^{2}\left( \Omega \right)$ with $s>1$, there exists $p_{M}\in M_{0,k-1}^{{\text {mod}}}\left( {\mathcal {T}} \right)$ such that

3.34 $$\begin{aligned} \left\| p-p_{M}\right\| _{L^{2}\left( {\mathbb {R}}^{2}\right) }\le C_{{\text {apx}}}\frac{\left( \left( 1+2\delta _{\max }\right) h_{{\mathcal {T}}}\right) ^{\min \left\{ k,s-1\right\} }}{k^{s-1}}\left\| p\right\| _{H^{s-1}\left( \Omega \right) } \end{aligned}$$

holds with $C_{{\text {apx}}}:=C\sqrt{{\text {card}}{{\mathcal {S}}}{{\mathcal {C}}}_{{\mathcal {T}}} }C_{{\text {Stein}}}C_{J}T_{k-1}\left( 1+c\delta _{\max }\right)$ and $\delta _{\max }$ from (3.32). The constant *C* depends only on $C_{{\text {ov}}}$ from (3.31) and on the shape regularity of the mesh.

An auxiliary result on the decomposition of functions ${\widetilde{Q}}_{0}^{h,k}$ from (3.14) precedes the proof of Theorem 3.14 below.

#### Lemma 3.15

Let all super critical vertices ${\textbf{z}} \in {{\mathcal {S}}}{{\mathcal {C}}}_{{\mathcal {T}}}$ be of Robinson type and let $k\ge 4$ be given. Then for all ${\widetilde{q}}\in {\widetilde{Q}}_{0}^{h,k}$ there exists $q\in M_{0,k-1}\left( {\mathcal {T}}\right)$ and $\theta _{{\textbf{z}}}\in {\mathbb {R}}$ for all ${\textbf{z}}\in {{\mathcal {S}}}{{\mathcal {C}}}_{{\mathcal {T}}}$ such that ${\tilde{q}}$ can be written as

$$\begin{aligned} {\widetilde{q}}=q+\sum _{{\textbf{z}}\in {{\mathcal {S}}}{{\mathcal {C}}}_{{\mathcal {T}}}}\theta _{{\textbf{z}}}\left( b_{k-1,{\textbf{z}}}\right) _{{\text {mvz}}}. \end{aligned}$$

#### Proof

Let ${\widetilde{q}}\in {\widetilde{Q}}_{0}^{h,k}\subset {\mathbb {P}}_{k-1,0}({\mathcal {T}})$ be arbitrarily chosen but fixed. The orthogonal decomposition of ${\mathbb {P}}_{k-1,0}({\mathcal {T}})$ in Proposition 3.5 provides $q\in M_{0,k-1}\left( {\mathcal {T}}\right)$ and $\theta _{{\textbf{z}}}\in {\mathbb {R}}$ for all ${\textbf{z}}\in {\mathcal {C}}_{{\mathcal {T}}}$ such that

$$\begin{aligned} {\widetilde{q}}=q+\sum _{{\textbf{z}}\in {\mathcal {C}}_{{\mathcal {T}}}}\theta _{{\textbf{z}}}\left( b_{k-1,{\textbf{z}}}\right) _{{\text {mvz}}}. \end{aligned}$$

For ${\textbf{z}}\in {\mathcal {C}}_{{\mathcal {T}}}{\setminus }{{\mathcal {S}}}{{\mathcal {C}}}_{{\mathcal {T}} }$, we locally decompose ${\mathbb {P}}_{k-1}\left( {\mathcal {T}}_{{\textbf{z}} }\right)$ into

$$\begin{aligned} {\mathbb {P}}_{k-1}\left( {\mathcal {T}}_{{\textbf{z}}}\right) =M_{0,k-1}\left( {\mathcal {T}}_{{\textbf{z}}}\right) \oplus {\text {span}}\left\{ b_{k-1,{\textbf{z}}}\right\} , \end{aligned}$$

where $M_{0,k-1}\left( {\mathcal {T}}_{{\textbf{z}}}\right) :=\left\{ \left. q\in {\mathbb {P}}_{k-1}\left( {\mathcal {T}}_{{\textbf{z}}}\right) \;\right| \;A_{{\mathcal {T}},{\textbf{z}}}\left( q\right) =0\right\}$. Since ${\tilde{q}}$ is continuous at ${\textbf{z}}$ and $N_{{\textbf{z}}}$ is even by ${\textbf{z}}\in {\mathcal {C}}_{{\mathcal {T}}}\setminus {{\mathcal {S}}}{{\mathcal {C}}}_{{\mathcal {T}}}$, the term $A_{{\mathcal {T}},{\textbf{z}}}\left( q\right) =0$ vanishes and $\left. q\right| _{\omega _{{\textbf{z}}}}\in M_{0,k-1}\left( {\mathcal {T}}_{{\textbf{z}}}\right)$ follows. This and $A_{{\mathcal {T}},{\textbf{z}}}\left( \left( b_{k-1,{\textbf{z}}}\right) _{{\text {mvz}} }\right) \ne 0$ from (3.16d) implies $\theta _{{\textbf{z}}}=0$ concluding the proof. $\square$

This lemma enables the proof of the approximation property for the modified pressure space.

#### Proof of Theorem 3.14

Let $p\in H^{s-1}\left( \Omega \right) \cap L_{0}^{2}\left( \Omega \right)$ be given. With a slight abuse of notation its extension by ${\mathcal {E}}_{{\text {Stein}}}$ to ${\mathbb {R}}^{2}$ is again denoted by *p*. Theorem 2.1 from [1] provides some $p_{Q}\in {\widetilde{Q}}_{0}^{h,k}\left( {\mathcal {T}}\right)$ that satisfies, for any $K\in {\mathcal {T}}$, the estimate

3.35 $$\begin{aligned} \left\| p-p_{Q}\right\| _{L^{2}\left( K\right) }\le C\frac{h_{K}^{\min \left\{ k,s-1\right\} }}{k^{s-1}}\left\| p\right\| _{H^{s-1}\left( \omega \left( K\right) \right) }. \end{aligned}$$

Lemma 3.15 reveals, for some $q\in M_{0,k-1}\left( {\mathcal {T}}\right)$, $C_0 \in {\mathbb {R}}$ and $\theta _{{\textbf{z}} }\in {\mathbb {R}}$ for all ${\textbf{z}}\in {{\mathcal {S}}}{{\mathcal {C}}}_{{\mathcal {T}}}$, the form

3.36 $$\begin{aligned} p_{Q}=q +\sum _{{\textbf{z}}\in {{\mathcal {S}}}{{\mathcal {C}}}_{{\mathcal {T}}}}\theta _{{\textbf{z}} }\left( b_{k-1,{\textbf{z}}}\right) _{{\text {mvz}}} = q + C_{0}+ \sum _{{\textbf{z}}\in {{\mathcal {S}}}{{\mathcal {C}}}_{{\mathcal {T}}}}\theta _{{\textbf{z}}}b_{k-1,{\textbf{z}} }. \end{aligned}$$

Triangle inequalities result with $p_{M}:=q+\sum _{{\textbf{z}}\in {{\mathcal {S}}}{{\mathcal {C}}}_{{\mathcal {T}}}}f_{{\textbf{z}}}\left( q\right) \left( b_{k-1,{\textbf{z}}}\right) _{{\text {mvz}}}\in M_{0,k-1}^{{\text {mod}}} \left( {\mathcal {T}} \right)$ in

3.37 $$\begin{aligned} \left\| p_{Q}-p_{M}\right\| _{L^{2}\left( \Omega \right) }\le \sum _{{\textbf{z}}\in {{\mathcal {S}}}{{\mathcal {C}}}_{{\mathcal {T}}}}\left| \theta _{{\textbf{z}} }-f_{{\textbf{z}}}\left( q\right) \right| \left\| b_{k-1,{\textbf{z}} }\right\| _{L^{2}\left( \Omega \right) }. \end{aligned}$$

Since the super-critical vertices ${\textbf{z}} \in {{\mathcal {S}}}{{\mathcal {C}}}_{{\mathcal {T}}}$ are of Robinson type and Assumption 3.10 holds, (3.36) and Remark 3.12(ii) reveal $q\vert _{K_{{\textbf{z}}}^{\prime }}=p_{Q}\vert _{K_{{\textbf{z}}}^{\prime }}-C_{0}$. Hence, (3.27) and algebraic manipulations result in

$$\begin{aligned} \left| \theta _{{{\textbf {z}}}}-f_{{{\textbf {z}}}}\left( q_{0}\right) \right|&=\left| \theta _{{{\textbf {z}}}}-\left( J_{{{\textbf {z}}}}\left( \left. p_{Q}\right| _{K_{{{\textbf {z}}}}^{\prime }}^{{\text {ext}} }-C_{0}\right) -J_{{{\textbf {z}}}}\left( \left. \left( p_{Q}-\theta _{{{\textbf {z}}}}b_{k-1,{{\textbf {z}}}}\right) \right| _{K_{{{\textbf {z}}}} }^{{\text {ext}}}-C_{0}\right) \right) \right| \\  &=\left| \theta _{{{\textbf {z}}}}-J_{{{\textbf {z}}}}\left( \left. p_{Q}\right| _{K_{{{\textbf {z}}}}^{\prime }}^{{\text {ext}}}\right) +J_{{{\textbf {z}}}}\left( \left. p_{Q}\right| _{K_{{{\textbf {z}}}} }^{{\text {ext}}}\right) -\theta _{{{\textbf {z}}}}J_{{{\textbf {z}}}}\left( \left. b_{k-1,{{\textbf {z}}}}\right| _{K_{{{\textbf {z}}}}}^{{\text {ext}} }\right) \right| \\  &\overset{(3.28)}{=}\quad \left| J_{{{\textbf {z}}}}\left( \left. p_{Q}\right| _{K_{{{\textbf {z}}}}}^{{\text {ext}}}-\left. p_{Q}\right| _{K_{{{\textbf {z}}}}^{\prime }}^{{\text {ext}}}\right) \right| . \end{aligned}$$

This, the bound (3.28) of the functional $J_{{\textbf{z}}}$ in Assumption 3.13, and triangle inequalities imply

$$\begin{aligned} C_{{{\textbf {z}}}}^{-1}&\left\| b_{k-1,{{\textbf {z}}}}\right\| _{L^{2}\left( \omega _{{{\textbf {z}}}}\right) }\left| \theta _{{{\textbf {z}}}}-f_{{{\textbf {z}}}}\left( q_{0}\right) \right| \le \left\| p_{Q}-\left. p_{Q}\right| _{K_{{{\textbf {z}}}}^{\prime }}^{{\text {ext}}}\right\| _{L^{2}\left( {\mathcal {U}}_{{{\textbf {z}}}}\right) }+\left\| \left. p_{Q}\right| _{K_{{{\textbf {z}}}}}^{{\text {ext}}}-p_{Q}\right\| _{L^{2}\left( {\mathcal {U}}_{{{\textbf {z}}}}\right) }\\  &\le 2\left\| p_{Q} -p\right\| _{L^{2}\left( K_{{{\textbf {z}}}}\cup K_{{{\textbf {z}}}}^{\prime }\right) }+\left\| \left. p_{Q}\right| _{K_{{{\textbf {z}}}} }^{{\text {ext}}}-p\right\| _{L^{2}\left( K_{{{\textbf {z}}} }^{{\text {ext}}}\right) }+\left\| \left. p_{Q}\right| _{K_{{{\textbf {z}}}}^{\prime }}^{{\text {ext}}}-p\right\| _{L^{2}\left( K_{{{\textbf {z}}}}^{\prime ,{\text {ext}}}\right) } \end{aligned}$$

with ${\mathcal {U}}_{{\textbf{z}}} \subseteq (K_{{\textbf{z}}} \cup K_{{\textbf{z}}}^{\prime })\cap K_{{\textbf{z}}}^{{\text {ext}}}\cap K_{{\textbf{z}}}^{\prime ,{\text {ext}}}$ in the last step. Lemma A.3 with $\kappa {:}{=}\min \{k,s-1\}$ and the definition of the neighborhood ${\widetilde{\omega }}_{{\textbf{z}}}$ in (3.30) control the second summand by

$$\begin{aligned}&\left\| \left. p_{Q}\right| _{K_{{{\textbf {z}}}}}^{{\text {ext}} }-p\right\| _{L^{2}\left( K_{{{\textbf {z}}}}^{{\text {ext}}}\right) }\\  &\quad \le T_{k-1}\left( 1+c\delta _{\max }\right) \left( 2\frac{\left( \left( 1+2\delta _{\max }\right) h_{{{\textbf {z}}}}\right) ^{\kappa }}{k^{s-1}}\left\| p\right\| _{H^{s-1}\left( K_{{{\textbf {z}}} }^{{\text {ext}}}\right) } +\left\| p-p_{Q}\right\| _{L^{2}\left( K_{{{\textbf {z}}}}\right) }\right) \\  &\quad \le CT_{k-1}\left( 1+c\delta _{\max }\right) \frac{\left( \left( 1+2\delta _{\max }\right) h_{{{\textbf {z}}}}\right) ^{\kappa }}{k^{s-1}}\left\| p\right\| _{H^{s-1}\left( {\widetilde{\omega }}_{{{\textbf {z}}}} \right) }, \end{aligned}$$

with the approximation property (3.35) of $p_Q$ in the last step. The previous two estimates, the analogous estimate for $\left\| \left. p_{Q}\right| _{K_{{\textbf{z}} }^{\prime }}^{{\text {ext}}}-p\right\| _{L^{2}\left( K_{{\textbf{z}} }^{\prime , {\text {ext}}}\right) }$ with the same upper bound, and (3.35) result in

$$\begin{aligned} \left| \theta _{{\textbf{z}}}-f_{{\textbf{z}}}\left( q_{0}\right) \right| \le CC_{{\textbf{z}}}T_{k-1}\left( 1+c\delta _{\max }\right) \frac{\left( \left( 1+2\delta _{\max }\right) h_{{\textbf{z}}}\right) ^{\kappa }}{k^{s-1}\left\| b_{k-1,{\textbf{z}}}\right\| _{L^{2}\left( \omega _{{\textbf{z}}}\right) }}\left\| p\right\| _{H^{s-1}\left( {\widetilde{\omega }}_{{\textbf{z}}}\right) }. \end{aligned}$$

Hence, (3.37) and $C_{{\textbf{z}}}\le C_J$ by definition in (3.32) verify

$$\begin{aligned} \left\| p_{Q}-p_{M}\right\| _{L^{2}\left( \Omega \right) }&\le CC_{J}T_{k-1}\left( 1+c\delta _{\max }\right) \frac{\left( \left( 1+2\delta _{\max }\right) h_{{\mathcal {T}}}\right) ^{\kappa }}{k^{s-1}} \sum _{{\textbf{z}}\in {{\mathcal {S}}}{{\mathcal {C}}}_{{\mathcal {T}}}}\left\| p\right\| _{H^{s-1}\left( {\widetilde{\omega }}_{{\textbf{z}}}\right) }. \end{aligned}$$

A Cauchy inequality in $\ell ^2$ and the finite overlay (3.31) of the regions ${\widetilde{\omega }}_{{\textbf{z}}}$ implies

$$\begin{aligned}&\left\| p_{Q}-p_{M}\right\| _{L^{2}\left( \Omega \right) }\\  &\quad \le CC_{J}T_{k-1}\left( 1+c\delta _{\max }\right) \sqrt{{\text {card}} {{\mathcal {S}}}{{\mathcal {C}}}_{{\mathcal {T}}}}\frac{\left( \left( 1+2\delta _{\max }\right) h_{{\mathcal {T}}}\right) ^{\kappa }}{k^{s-1}}\left\| p\right\| _{H^{s-1}\left( {\mathbb {R}}^{2}\right) }. \end{aligned}$$

This and $\Vert p\Vert _{H^{s-1}({\mathbb {R}}^2)}\le C_{{\text {Stein}}}\Vert p\Vert _{H^{s-1}(\Omega )}$ from the Stein extension (cf., (3.33) conclude the proof. $\square$

### Recovery of optimal rates for the postprocessed pressure

The structural assumptions on the functional $f_{{\textbf{z}}}$ in Assumption 3.13 for optimal approximation properties of the resulting modified pressure space $M_{0,k-1}^{{\text {mod}} }\left( {\mathcal {T}}\right)$ in Theorem 3.14 leave considerable freedom in the particular choice of the functionals $f_{{\textbf{z}}}$. Below we give two examples.

#### Example 3.16

Two possible choices of $J_{{\textbf{z}}}$ and $f_{{\textbf{z}}}$ are given by

1. the point evaluation at ${\textbf{z}}\in {\mathcal {S}}{\mathcal {C}}_{{\mathcal {T}}}$, namely
  3.38 $$\begin{aligned} J_{{\textbf{z}}}\left( q\right) :=\frac{q\left( {\textbf{z}}\right) }{\left. b_{k-1,{\textbf{z}}}\right| _{K_{{\textbf{z}}}}\left( {\textbf{z}}\right) }\quad \forall q\in {\mathbb {P}}_{k-1}\left( {\mathbb {R}}^{2}\right) , \end{aligned}$$
  so that
  3.39 $$\begin{aligned} f_{{\textbf{z}}}\left( q\right) =\frac{\left( \left. q\right| _{K_{{\textbf{z}}}^{\prime }}^{{\text {ext}}}-\left. q\right| _{K_{{\textbf{z}}}}\right) \left( {\textbf{z}}\right) }{\left. b_{k-1,{\textbf{z}}}\right| _{K_{{\textbf{z}}}}\left( {\textbf{z}}\right) }\quad \forall q\in M_{0,k-1}\left( {\mathcal {T}}\right) , \end{aligned}$$
2. the integration with weight $b_{k-1,{\textbf{z}}}\vert _{K_{{\textbf{z}}}}^{{\text {ext}}}$ over a subset $S_{{\textbf{z}}}^{\prime }\subset K_{{\textbf{z}}}^{\prime }$ with positive measure, namely
  $$\begin{aligned} J_{{\textbf{z}}}\left( q\right) :=\frac{\int _{S_{{\textbf{z}}}^{\prime }}q\left. b_{k-1,{\textbf{z}}}\right| _{K_{{\textbf{z}}}}^{{\text {ext}}} }{\left\| \left. b_{k-1,{\textbf{z}}}\right| _{K_{{\textbf{z}}} }^{{\text {ext}}}\right\| _{L^{2}\left( S_{{\textbf{z}}}^{\prime }\right) }^{2}}\quad \forall q\in {\mathbb {P}}_{k-1}\left( {\mathbb {R}} ^{2}\right) , \end{aligned}$$
  so that
  3.40 $$\begin{aligned} f_{{\textbf{z}}}\left( q\right) =\frac{\int _{S_{{\textbf{z}}}^{\prime }}\left( q-\left. q\right| _{K_{{\textbf{z}}}}^{{\text {ext}}}\right) \left. b_{k-1,{\textbf{z}}}\right| _{K_{{\textbf{z}}}}^{{\text {ext}}} }{\left\| \left. b_{k-1,{\textbf{z}}}\right| _{K_{{\textbf{z}}} }^{{\text {ext}}}\right\| _{L^{2}\left( S_{{\textbf{z}}}^{\prime }\right) }^{2}}\quad \forall q\in M_{0,k-1}\left( {\mathcal {T}}\right) . \end{aligned}$$

The implementation of the first choice is simple and its numerical evaluation very fast. However, for high polynomial degree *k* the polynomial extrapolation, possibly, becomes increasingly numerically unstable and the second choice might be preferable for such cases.

Next, we prove optimal convergence rates with respect to the mesh width of the postprocessed pressure $p_{M}^{*}$ from (3.20) for the functional $J_{{\textbf{z}}}$ in Example 3.16(1) so that $f_{{\textbf{z}}}$ is given by (3.39).

#### Lemma 3.17

Let Assumption 3.10 hold and let the functionals $J_{{\textbf{z}}}$ and $f_{{\textbf{z}}}$ be defined by (3.38) and (3.39). Then, Assumption 3.13 is satisfied.

#### Proof

Proposition 3.4, (3.38), and an inverse inequality (see, e.g., [20]) verify for any $q\in {\mathbb {P}}_{k-1}\left( {\mathbb {R}}^{2}\right)$ and ${\textbf{z}}\in {{\mathcal {S}}}{{\mathcal {C}}}_{{\mathcal {T}}}$ that

$$\begin{aligned} \left| J_{{\textbf{z}}}\left( q\right) \right| =\frac{\left| q\left( {\textbf{z}}\right) \right| }{\left| \left. b_{k-1,{\textbf{z}} }\right| _{K_{{\textbf{z}}}}\left( {\textbf{z}}\right) \right| } =\frac{\left| q\left( {\textbf{z}}\right) \right| }{\left( {\begin{array}{c}k+1\\ 2\end{array}}\right) \left| K_{{\textbf{z}}}\right| ^{-1}}\le 2\frac{h_{{\textbf{z}}}^{2} }{k^{2}}\left\| q\right\| _{L^{\infty }\left( K_{{\textbf{z}}}\right) }\le C_{{\text {inv}}} h_{{\textbf{z}}}\left\| q\right\| _{L^{2}\left( K_{{\textbf{z}}}\right) }, \end{aligned}$$

where $C_{{\text {inv}}} >0$ is independent of $h_{{\textbf{z}}}$ and *k*. Next, we use (3.16b) to obtain

$$\begin{aligned} \left\| b_{k-1,{\textbf{z}}}\right\| _{L^{2}\left( \omega _{{\textbf{z}} }\right) }^{2}=\sum _{K\in {\mathcal {T}}_{{\textbf{z}}}}\left| K\right| ^{-1}\le C_{{\text {sr}}}^{2}h_{{\textbf{z}}}^{-2} \end{aligned}$$

for a constant $C_{{\text {sr}}}$ depending only on the shape regularity of the mesh. This implies the continuity,

$$\begin{aligned} \left| J_{{\textbf{z}}}\left( q\right) \right| \le \frac{C_{{\text {inv}}} C_{{\text {sr}}}}{\left\| b_{k-1,{\textbf{z}} }\right\| _{L^{2}\left( \omega _{{\textbf{z}}}\right) }}\left\| q\right\| _{L^{2}\left( K_{{\textbf{z}}}\right) }. \end{aligned}$$

By setting ${\mathcal {U}}_{{\textbf{z}}}:=K_{{\textbf{z}}}$ we see that (3.28) is satisfied for $C_{{\textbf{z}}}:=C_{0}C_{1}$. We choose $K_{{\textbf{z}}}^{{\text {ext}}}:=K_{{\textbf{z}}}$ and $K_{{\textbf{z}}}^{\prime ,{\text {ext}}}$ as a minimal triangle which contains $K_{{\textbf{z}}}\cup K_{{\textbf{z}}}^{\prime }$ so that (3.29) holds for a constant $\delta _{{\textbf{z}}}=O\left( 1\right)$ which only depends on the shape regularity of the mesh. It remains to notice that the definition (3.39) is of the form (3.27). $\square$

A combination of Theorems 3.7 and 3.14 implies optimal convergence rates in terms of the mesh width $h_{{\mathcal {T}}}$ of our simple postprocessing for the Scott–Vogelius element.

#### Theorem 3.18

Let Assumption 3.10 hold and let the functionals $J_{{\textbf{z}}}$ and $f_{{\textbf{z}}}$ be defined by (3.38) and (3.39). Let the assumptions in Theorem 3.7 be satisfied. Then there exists a constant $C>0$ depending on the shape-regularity of ${\mathcal {T}}$, the constants $C_{{\text {Stein}}}$, $C_{{\text {ov}}}$, the domain $\Omega$, and the polynomial degree $k\ge 4$ such that the postprocessed pressure $p_{M}^{*}$ from (3.20) satisfies

$$\begin{aligned} \left\| p-p_{M}^{*}\right\| _{L^{2}\left( \Omega \right) }\le Ch_{{\mathcal {T}}}^{\min \left\{ k,s-1\right\} }\left\| p\right\| _{H^{s-1}\left( \Omega \right) }. \end{aligned}$$

## Pressure-improvement for the pressure-wired Stokes element

The pressure-wired Stokes element introduced in [13] generalises the standard Scott–Vogelius element by restricting the discrete pressure space additionally at nearly singular vertices ${\textbf{z}}\in {\mathcal {V}}\left( {\mathcal {T}}\right)$ *(called* $\eta$*-critical vertices)* to guarantee a mesh-robust inf-sup stability. In general, the divergence of the discrete velocity field no longer vanishes pointwise while its marginality has been analyzed in detail in [13, Sec. 5]. This section discusses a modified pressure space with a parameter $\eta \ge 0$ introduced below in full analogy to Sect. 3 for optimal approximation properties.

### Definition 4.1

For $\eta \ge 0$, the set of $\eta$*-critical vertices* is given by

4.1 $$\begin{aligned} {\mathcal {C}}_{{\mathcal {T}}}\left( \eta \right) :=\left\{ \left. {\textbf{z}} \in {\mathcal {V}}\left( {\mathcal {T}}\right) \;\right| \;\Theta \left( {\textbf{z}}\right) \le \eta \right\} \end{aligned}$$

and the subset of $\eta$*-super critical vertices* by

$$\begin{aligned} {{\mathcal {S}}}{{\mathcal {C}}}_{{\mathcal {T}}}\left( \eta \right) :=\left\{ \left. {\textbf{z}}\in {\mathcal {C}}_{{\mathcal {T}}}\left( \eta \right) \;\right| \;N_{{\textbf{z}}}=1,3\right\} . \end{aligned}$$

Definition 3.11 generalises to $\eta$-super critical vertices: a vertex $\mathbf {z\in }{{\mathcal {S}}}{{\mathcal {C}}}_{{\mathcal {T}}}\left( \eta \right)$ is called a *Robinson vertex* if (i): ${\mathcal {T}} _{{\textbf{z}}}^{*}\cap {\mathcal {T}}_{{\textbf{y}}}^{*}=\emptyset$ for all ${\textbf{y}}\in {{\mathcal {S}}}{{\mathcal {C}}}_{{\mathcal {T}}}\left( \eta \right) {\setminus }\left\{ {\textbf{z}}\right\}$ and (ii): $\omega _{{\textbf{z}}}^{*}\cap {\mathcal {C}} _{{\mathcal {T}}}\left( \eta \right) =\left\{ {\textbf{z}}\right\}$ hold.

### Remark 4.2

**Fig. 4:**
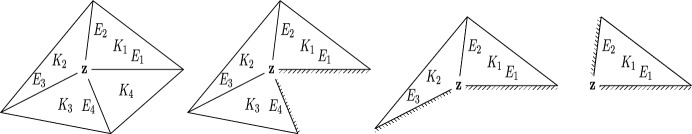
Vertex patch for an interior $\eta$-critical vertex ${\textbf{z}}\in {\mathcal {V}}_{\Omega }({\mathcal {T}})$ with $N_{{\textbf{z}} }=4$ (resp. boundery $\eta$-critical vertex ${\textbf{z}}\in {\mathcal {V}} _{\partial \Omega }({\mathcal {T}})$ with $N_{{\textbf{z}}}=1,2,3$) triangles

For sufficiently small $\eta _{0}$ (depending only on the shape regularity of the mesh and on $\Omega$) and $\eta \in [0,\eta _{0}[,$ only the four types of $\eta$-critical vertex configurations depicted in Fig. 4 are possible; see [19, Lem. 2.13] for details. Notice that ${\mathcal {C}}_{{\mathcal {T}}}\left( 0\right) ={\mathcal {C}}_{{\mathcal {T}}}$ and ${{\mathcal {S}}}{{\mathcal {C}}}_{{\mathcal {T}}}\left( 0\right) ={{\mathcal {S}}}{{\mathcal {C}}} _{{\mathcal {T}}}$ hold. Since ${\mathcal {C}}_{{\mathcal {T}}}(\eta ) = {\mathcal {C}}_{{\mathcal {T}}}(1)$ for all $\eta \ge 1$ from $\Theta ({\textbf{z}})\le 1$, $\eta \le 1$ in the following is not a restriction.

### Definition 4.3

*(pressure-wired Stokes element,* [13, Lem. 1]*)* Given $k\in {\mathbb {N}}$ and $0\le \eta \le 1$, the *pressure-wired Stokes element* $\left( {\textbf{S}}_{k,0}\left( {\mathcal {T}}\right) ,M_{\eta ,k-1}\left( {\mathcal {T}}\right) \right)$ is defined with the *reduced pressure space*

4.2 $$\begin{aligned} M_{\eta ,k-1}\left( {\mathcal {T}}\right) :=\left\{ \left. q\in {\mathbb {P}} _{k-1,0}\left( {\mathcal {T}}\right) \;\right| \;\forall {\textbf{z}} \in {\mathcal {C}}_{{\mathcal {T}}}\left( \eta \right) :\ A_{{\mathcal {T}},{\textbf{z}} }\left( q\right) =0\right\} . \end{aligned}$$

As already announced in the introduction, there is a significant loss in accuracy of the pressure approximation for the pressure-wired Stokes element if the mesh contains $\eta$-super critical vertices. As a remedy, we introduce a modification in complete analogy to Sect. 3 for the Scott–Vogelius element.

### Definition 4.4

*(modified pressure-wired Stokes element)* Given $k\in {\mathbb {N}}, 0\le \eta \le 1$, and functionals $f_{{\textbf{z}}}:{\mathbb {P}}_{k-1}\left( {\mathcal {T}}\right) \rightarrow {\mathbb {R}}$ with (3.18) for all ${\textbf{z}}\in {{\mathcal {S}}}{{\mathcal {C}}} _{{\mathcal {T}}}\left( \eta \right)$, the *modified pressure-wired Stokes element* $\left( {\textbf{S}}_{k,0}\left( {\mathcal {T}}\right) ,M_{\eta ,k-1} ^{{\text {mod}}}\left( {\mathcal {T}}\right) \right)$ is given by the modified reduced pressure space

4.3 $$\begin{aligned} M_{\eta ,k-1}^{{\text {mod}}}\left( {\mathcal {T}}\right) :=\left\{ q+\sum _{{\textbf{z}}\in {{\mathcal {S}}}{{\mathcal {C}}}_{{\mathcal {T}}}\left( \eta \right) }f_{{\textbf{z}}}\left( q\right) \left( b_{k-1,{\textbf{z}}}\right) _{{\text {mvz}}}:\;q\in M_{\eta ,k-1}\left( {\mathcal {T}}\right) \right\} . \end{aligned}$$

### Stability of the modified pressure-wired Stokes element

The modified pressure-wired Stokes element inherits the mesh-robust discrete inf-sup stability.

#### Lemma 4.5

The modified pressure-wired Stokes element is inf-sup stable for all $k\ge 4$ and $0\le \eta \le 1$ with $\beta \left( {\textbf{S}} _{k,0}\left( {\mathcal {T}}\right) ,M_{\eta ,k-1}^{{\text {mod}}}\left( {\mathcal {T}}\right) \right) \ge c \Theta _{\min } \left( \eta \right) / C_{f}$ for $C_f=1+\sum  _{{\textbf{z}}\in {\mathcal {S}}{\mathcal {C}}_{{\mathcal {T}}}(\eta )} C_{f_{{\textbf{z}}}}$ and $\Theta _{\min } \left( \eta \right)$ from [13, Eqn. (14)] which generalizes the global measure of singularity in (3.8). The constant $c>0$ exclusively depends on the shape regularity of the mesh.

#### Proof

The proof of Lemma 4.5 is a modification of that of Lemma 3.9 and given for completeness. The surjective extrapolation operator ${\mathcal {E}}_{\eta ,k}:M_{\eta ,k-1}\left( {\mathcal {T}}\right) \rightarrow M_{\eta ,k-1}^{{\text {mod}}}\left( {\mathcal {T}}\right)$ given by

$$\begin{aligned} {\mathcal {E}}_{\eta ,k}q=q+\sum _{{\textbf{z}}\in {{\mathcal {S}}}{{\mathcal {C}}}_{{\mathcal {T}}}\left( \mathcal {\eta }\right) }f_{{\textbf{z}}}\left( q\right) \left( b_{k-1,{\textbf{z}}}\right) _{{\text {mvz}}}\qquad \forall q\in M_{\eta ,k-1}\left( {\mathcal {T}}\right) \end{aligned}$$

generalises ${\mathcal {E}}_{k}$ from (3.24). Lemma 2 in [13] provides, for any $q\in M_{\eta ,k-1}\left( {\mathcal {T}}\right)$, some ${\textbf{v}} _{q}:=\Pi _{k}q\in {\textbf{S}}_{k,0}({\mathcal {T}})$ with ${\text {div}}({\textbf{v}}_q)=q$ and $c \Theta _{\min } \left( \eta \right) \Vert {\textbf{v}}_q\Vert _{{\textbf{H}}^1(\Omega )}\le \Vert q\Vert _{L^2(\Omega )}$. As in the proof of Lemma 3.9,

$$\begin{aligned} \left( {\mathcal {E}}_{\eta ,k}q,q\right) _{L^{2}\left( \Omega \right) }=\Vert q\Vert _{L^2(\Omega )}^2\quad \text{ and }\quad \left\| {\mathcal {E}}_{\eta ,k}q\right\| _{L^{2}\left( \Omega \right) }\le C_f \left\| q\right\| _{L^{2}\left( \Omega \right) } \quad \forall q\in M_{\eta ,k-1}\left( {\mathcal {T}}\right) , \end{aligned}$$

the right-inverse of the divergence, and the surjectivity of ${\mathcal {E}}_{\eta ,k}$ bound the inf-sup constant by

$$\begin{aligned} \inf _{q^{*}\in M_{\eta ,k-1}^{{\text {mod}}}\left( {\mathcal {T}}\right) \setminus \left\{ 0\right\} }\sup _{{\textbf{v}}\in {\textbf{S}}_{k,0}\left( {\mathcal {T}}\right) \setminus \left\{ {\textbf{0}}\right\} }\frac{\left( q^{*},{\text {div}}{\textbf{v}}\right) _{L^{2}\left( \Omega \right) } }{\left\| q^{*}\right\| _{L^{2}\left( \Omega \right) }\left\| {\textbf{v}}\right\| _{H^{1}\left( \Omega \right) }}&\ge \inf _{q\in M_{\eta ,k-1}\left( {\mathcal {T}}\right) \setminus \left\{ 0\right\} } \frac{\left( {\mathcal {E}}_{\eta ,k}q,q\right) _{L^{2}\left( \Omega \right) }}{\left\| {\mathcal {E}}_{\eta ,k}q\right\| _{L^{2}\left( \Omega \right) }\left\| {\textbf{v}}_{q}\right\| _{H^{1}\left( \Omega \right) }}\\&\ge c \Theta _{\min } \left( \eta \right) /C_f . \end{aligned}$$

$$\square $$

A consequence of Lemma 4.5 and the theory of mixed methods [6, Chap. 5, Thm. 5.2.3] is the quasi-optimality of the discrete solution. Recall $C_f$ from Lemma 4.5.

#### Corollary 4.6

Given $k\ge 4$ and $0\le \eta \le 1$, let $\left( {\textbf{u}},p\right) \in {\textbf{H}}_{0}^{1}\left( \Omega \right) \times L_{0}^{2}\left( \Omega \right)$ solve (2.2). The discrete solution $\left( {\textbf{u}}_{S},p_{M}^{*}\right)$ of (2.3) for the choice $\left( {\textbf{S}},M\right) =\left( {\textbf{S}}_{k,0}\left( {\mathcal {T}}\right) ,M_{\eta ,k-1}^{{\text {mod}}}\left( {\mathcal {T}} \right) \right)$ satisfies the quasi-optimal error estimate

4.4a $$\begin{aligned} \left\| \nabla \left( {\textbf{u}}-{\textbf{u}}_{{\textbf{S}}} \right) \right\| _{{\mathbb {L}}^{2}\left( \Omega \right) }&\le C\left( \frac{C_f }{\Theta _{\min } \left( \eta \right) } \inf _{{\textbf{v}}\in {\textbf{S}} _{k,0}\left( {\mathcal {T}}\right) }\left\| {\textbf{u}}-{\textbf{v}}\right\| _{{\textbf{H}}^{1}\left( \Omega \right) }+\inf _{q\in M_{\eta ,k-1}^{{\text {mod}} }\left( {\mathcal {T}}\right) }\left\| p-q\right\| _{L^{2}\left( \Omega \right) }\right) , \end{aligned}$$

4.4b $$\begin{aligned} \left\| p-p_{M}^{*}\right\| _{L^{2}\left( \Omega \right) }&\le \frac{C_{{\text {vel}}}C_f^2}{ \Theta _{\min } \left( \eta \right) ^{2}}\inf _{{\textbf{v}}\in {\textbf{S}}_{k,0}\left( {\mathcal {T}}\right) }\left\| {\textbf{u}} -{\textbf{v}}\right\| _{{\textbf{H}}^{1}\left( \Omega \right) }+ \frac{C_{{\text {pres}}}C_f}{\Theta _{\min } \left( \eta \right) }\inf _{q\in M_{\eta ,k-1}^{{\text {mod}}}\left( {\mathcal {T}}\right) }\left\| p-q\right\| _{L^{2}\left( \Omega \right) }. \end{aligned}$$

The positive constants $C,C_{{\text {vel}}},C_{{\text {pres}}}$ only depend on the shape-regularity of the mesh and the domain $\Omega$.

### Optimal convergence rates

Convergence rates for the discrete solution $\left( {\textbf{u}}_{{\textbf{S}}},p_{M}^{*}\right)$ follow from Corollary 4.6 once the approximation property of $M_{\eta ,k-1}^{{\text {mod}}}\left( {\mathcal {T}}\right)$ is clarified. One key argument is an analog characterisation of the orthogonal complement of $M_{\eta ,k-1}({\mathcal {T}})$ in $\mathbb {P}_{k-1,0}({\mathcal {T}})$ as in Proposition 3.5.

#### Lemma 4.7

Let all $\eta$-super critical vertices be of Robinson type. Then the decomposition

$$\begin{aligned} {\mathbb {P}}_{k-1,0}({\mathcal {T}}) = M_{\eta ,k-1}({\mathcal {T}})&\oplus {\text {span}}\{b_{k-1,{\textbf{z}}}\ |\ {\textbf{z}}\in {\mathcal {C}}_{{\mathcal {T}}}(\eta )\setminus {\mathcal {S}}{\mathcal {C}}_{{\mathcal {T}}}(\eta )\}\\&\oplus {\text {span}}\{(b_{k-1,{\textbf{y}}})_{{\text {mvz}}}\ |\ {\textbf{y}}\in {\mathcal {S}}{\mathcal {C}}_{{\mathcal {T}}}(\eta )\} \end{aligned}$$

is $L^2$ orthogonal, i.e., any ${\textbf{z}}\in {\mathcal {C}}_{{\mathcal {T}}}(\eta ){\setminus }{\mathcal {S}}{\mathcal {C}}_{{\mathcal {T}}}(\eta )$, ${\textbf{y}}\in {\mathcal {S}}{\mathcal {C}}_{{\mathcal {T}}}(\eta )$, and $q_M\in M_{\eta ,k-1}({\mathcal {T}})$ satisfy

$$\begin{aligned} (q_M, b_{k-1,{{\textbf {z}}}})_{L^2(\Omega )}=\left( q_M, \left( b_{k-1,{{\textbf {y}}}}\right) _{{\text {mvz}}}\right) _{L^2(\Omega )}=\left( b_{k-1,{{\textbf {z}}}}, \left( b_{k-1,{{\textbf {y}}}}\right) _{{\text {mvz}}}\right) _{L^2(\Omega )}=0. \end{aligned}$$

#### Proof

Since $N_{{\textbf{z}}}$ is even for all ${\textbf{z}}\in {\mathcal {C}}_{{\mathcal {T}}}(\eta )\setminus {\mathcal {S}}{\mathcal {C}}_{{\mathcal {T}}}(\eta )$ by definition, (3.16a) in Proposition 3.4 verifies $\overline{b_{k-1,{\textbf{z}}}}=0$ so that $b_{k-1,{\textbf{z}}}=\left( b_{k-1,{\textbf{z}}}\right) _{{\text {mvz}}}$. This, the orthogonality $(q_M, \left( b_{k-1,{\textbf{y}}}\right) _{{\text {mvz}}})_{L^2(\Omega )}$ for all $q_M\in M_{\eta ,k-1}({\mathcal {T}})$ and ${\textbf{z}}\in {\mathcal {C}}_{{\mathcal {T}}}(\eta )$ as in Proposition 3.4, and Remark 3.12(i) adapted to $\eta$-critical vertices conclude the proof; further details are omitted. $\square$

As in Sect. 3, we employ a representation of the space ${\widetilde{Q}}_{0}^{h,k}$ with optimal approximation properties from (3.14) in terms of functions in $M_{\eta ,k-1}\left( {\mathcal {T}}\right)$ and $\left\{ \left. b_{k-1,{\textbf{z}}}\;\right| \;{\textbf{z}}\in {\mathcal {C}}_{{\mathcal {T}} }\left( \eta \right) \right\}$.

#### Lemma 4.8

Let all $\eta$-super critical vertices be of Robinson type and let $k\ge 4$ be given. Then for all ${\widetilde{q}} \in {\widetilde{Q}}_{0}^{h,k}$ there exist $q_{0}\in M_{\eta ,k-1}\left( {\mathcal {T}}\right)$ and $\theta _{{\textbf{z}}}\in {\mathbb {R}}$ for all ${\textbf{z}}\in {{\mathcal {S}}}{{\mathcal {C}}}_{{\mathcal {T}}}\left( \eta \right)$ such that

4.5 $$\begin{aligned} {\widetilde{q}} =q_{0}+\sum _{{\textbf{z}}\in {{\mathcal {S}}}{{\mathcal {C}}}_{{\mathcal {T}}}\left( \eta \right) }\theta _{{\textbf{z}}}\left( b_{k-1,{\textbf{z}}}\right) _{{\text {mvz}}}. \end{aligned}$$

#### Proof

The proof is a straightforward modification of that of Proposition 3.15 with the orthogonal decomposition of ${\mathbb {P}}_{k-1,0}({\mathcal {T}})$ from Lemma 4.7; further details are omitted. $\square$

#### Theorem 4.9

Let all $\eta$-super critical vertices be of Robinson type and let Assumption 3.13 be satisfied. For any $p\in H^{s-1}\left( \Omega \right) \cap L_{0}^{2}\left( \Omega \right)$ with $s>1$, there exists $p_{M}^{*}\in M_{\eta ,k-1}^{{\text {mod}} }\left( {\mathcal {T}}\right)$ such that

4.6 $$\begin{aligned} \left\| p-p_{M}^{*}\right\| _{L^{2}\left( \Omega \right) }\le C_{{\text {apx}}}\frac{\left( \left( 1+2\delta _{\max }\right) h_{{\mathcal {T}}}\right) ^{\min \left\{ k,s-1\right\} }}{k^{s-1}}\left\| p\right\| _{H^{s-1}\left( \Omega \right) } \end{aligned}$$

for $C_{{\text {apx}}}:=C\sqrt{{\text {card}}{{\mathcal {S}}}{{\mathcal {C}}}_{{\mathcal {T}} }\left( \eta \right) }C_{{\text {Stein}}}C_{J}T_{k-1}\left( 1+c\delta _{\max }\right)$ with $C_{J}$ from (3.32) and $\delta _{\max }$ from (3.32). The constant *C* depends only on $C_{{\text {ov}}}$ from (3.31) and on the shape regularity of the mesh.

The proof of this theorem follows by applying the arguments in the proof of Theorem 3.14 verbatim to ${{\mathcal {S}}}{{\mathcal {C}}} _{{\mathcal {T}}}\left( \eta \right)$ and Lemma 4.8 instead of ${{\mathcal {S}}}{{\mathcal {C}}} _{{\mathcal {T}}}$ and Lemma 3.15.

### Control of ${\text {div}}{\textbf{u}}_{{\textbf{S}}}$

The pressure-wired Stokes element reduces the pressure space not only at exactly singular vertices ${\mathcal {C}}_{{\mathcal {T}}}$ but also at $\eta$-critical vertices ${\textbf{z}}\in {\mathcal {C}}_{{\mathcal {T}}}\left( \eta \right) \backslash {\mathcal {C}}_{{\mathcal {T}}}$. Therefore, the discrete velocity field ${\textbf{u}}_{{\textbf{S}}}\in {\textbf{S}}_{k,0}\left( {\mathcal {T}}\right)$ of the pressure-wired Stokes element is in general not pointwise divergence free. However, Theorem 3 in [13] guarantees that $\left\| {\text {div}}{\textbf{u}}_{{\textbf{S}} }\right\| _{L^{2}\left( \Omega \right) }$ tends to zero at least linearly in $\eta$. An analogous statement holds for the modified pressure-wired Stokes element. Consider the open subset

4.7 $$\begin{aligned} \Omega (\eta ):= \bigcup _{{{\textbf {z}}}\in {\mathcal {C}}_{{\mathcal {T}}}(\eta )}\overset{\circ }{\omega }\ _{{\textbf {z}}}\subset \Omega \end{aligned}$$

for $0\le \eta \le 1$, where $\overset{\circ }{\omega }\ _{{\textbf {z}}}$ denotes the interior of the vertex patch $\omega _{{\textbf{z}}}$ from (3.1), and define

4.8 $$\begin{aligned} {\textbf{S}}_{\eta ,k,0}\left( {\mathcal {T}}\right) :=\left\{ {\textbf{v}} \in {\textbf{S}}_{k,0}\left( {\mathcal {T}}\right) \mid A_{{\mathcal {T}},{\textbf{z}} }\left( {\text {div}}{\textbf{v}}\right) =0\quad \forall {\textbf{z}} \in {\mathcal {C}}_{{\mathcal {T}}}\left( \eta \right) \right\} . \end{aligned}$$

#### Theorem 4.10

There exists $\eta _0>0$ such that the following holds: Given $0\le \eta < \eta _0$, let Assumption 3.13 be satisfied for all $\eta$-super critical vertices ${\textbf{z}}\in {\mathcal {S}}{\mathcal {C}}_{{\mathcal {T}}}(\eta )$ that are additionally assumed to be of Robinson type. For $k\ge 4$, the discrete solution $\left( {\textbf{u}}_{{\textbf{S}}},p_{M}^{*}\right) \in \left( {\textbf{S}}_{k,0}\left( {\mathcal {T}}\right) ,M_{\eta ,k-1} ^{{\text {mod}}}\left( {\mathcal {T}}\right) \right)$ to (2.3) satisfies

4.9 $$\begin{aligned} \left\| {\text {div}}{\textbf{u}}_{{\textbf{S}}}\right\| _{L^{2}\left( \Omega \right) }\le C_{{\text {div}}}\eta \inf _{{\textbf{w}}_{{\textbf{S}} }\in {\textbf{S}}_{\eta ,k,0}\left( {\mathcal {T}}\right) }\left\| \nabla \left( {\textbf{u}}_{{\textbf{S}}}-{\textbf{w}}_{{\textbf{S}}}\right) \right\| _{{\mathbb {L}}^{2}\left( \Omega (\eta )\right) }. \end{aligned}$$

The constant $C_{\textrm{div}}>0$ in (4.9) only depends on the shape-regularity constant and the domain $\Omega$.

The remaining parts of this section are devoted to the proof of Theorem 4.10. Since ${\textbf{u}}_{{\textbf{S}}}$ solves (2.3) and has integral mean zero, its divergence is orthogonal to ${M_{\eta ,k-1}^{{\text {mod}} }\left( {\mathcal {T}}\right) }$ in $P_{k-1,0}({\mathcal {T}})$, namely

4.10 $$\begin{aligned} {\text {div}}{\textbf{u}}_{{\textbf{S}}}\in {M_{\eta ,k-1}^{{\text {mod}} }\left( {\mathcal {T}}\right) }^{\perp }:=\left\{ \left. q\in {\mathbb {P}} _{k-1,0}\left( {\mathcal {T}}\right) \;\right| \;\forall p\in M_{\eta ,k-1}^{{\text {mod}}}\left( {\mathcal {T}}\right) :\ \left( q,p\right) _{L^{2}\left( \Omega \right) }=0\right\} . \end{aligned}$$

We characterise the $L^{2}\left( \Omega \right)$-orthogonal complement $M_{\eta ,k-1}^{{\text {mod}}}\left( {\mathcal {T}}\right) ^{\perp }$ to prove Theorem 4.10. Let $\phi _{{\textbf{z}}}\in M_{\eta ,k-1}\left( {\mathcal {T}}\right)$ be the Riesz representative of $f_{{\textbf{z}}}:M_{\eta ,k-1}\left( {\mathcal {T}}\right) \rightarrow {\mathbb {R}}$, i.e., $\phi _{{\textbf{z}}}$ satisfies

4.11 $$\begin{aligned} \left( \phi _{{\textbf{z}}},q_{0}\right) _{L^{2}\left( \Omega \right) }=f_{{\textbf{z}}}\left( q_{0}\right) \qquad \forall q_{0}\in M_{\eta ,k-1}\left( {\mathcal {T}}\right) . \end{aligned}$$

#### Lemma 4.11

Under the assumptions of Theorem 4.10, a basis for $M_{\eta ,k-1}^{{\text {mod}}}\left( {\mathcal {T}}\right) ^{\perp }={\text {span}}\left( {\mathcal {B}}\right)$ is given by ${\mathcal {B}}:=\left\{ \left. b_{k-1,{\textbf{z}}}\;\right| \;\forall {\textbf{z}}\in {\mathcal {C}} _{{\mathcal {T}}}\left( \eta \right) {\setminus }{{\mathcal {S}}}{{\mathcal {C}}}_{{\mathcal {T}}}\left( \eta \right) \right\} \cup \left\{ \left. \varphi _{{\textbf{z}}}\;\right| \;\forall {\textbf{z}}\in {{\mathcal {S}}}{{\mathcal {C}}}_{{\mathcal {T}}}\left( \eta \right) \right\}$ with

4.12 $$\begin{aligned} \varphi _{{\textbf{z}}}:=\phi _{{\textbf{z}}}-\frac{1}{\left\| b_{k-1,{\textbf{z}}}\right\| _{L^{2}\left( \Omega \right) }^{2}}\left( \left( b_{k-1,{\textbf{z}}}\right) _{{\text {mvz}}} +\sum _{{\textbf{y}}\in {{\mathcal {S}}}{{\mathcal {C}}}_{{\mathcal {T}}}\left( \eta \right) }\left( \overline{b_{k-1,{\textbf{z}}}},b_{k-1,{\textbf{y}}}\right) _{L^{2}\left( \Omega \right) }\phi _{{\textbf{y}}}\right) . \end{aligned}$$

#### Proof

A counting argument with Propostion 3.4 and Lemma 4.7 show

$$\begin{aligned} {\text {card}} {\mathcal {B}} = {\text {card}} {\mathcal {C}}_{{\mathcal {T}}} \left( \eta \right) ={\text {dim}} {M_{\eta ,k-1}\left( {\mathcal {T}}\right) }^{\perp }, \end{aligned}$$

where the orthogonal complement ${M_{\eta ,k-1}\left( {\mathcal {T}}\right) }^{\perp }$ of ${M_{\eta ,k-1}\left( {\mathcal {T}}\right) }$ in ${\mathbb {P}}_{k-1,0}({\mathcal {T}})$ is defined analogously to (4.10). The definition of $M_{\eta ,k-1}^{{\text {mod}}}\left( {\mathcal {T}}\right)$ in (4.3) and $\left( b_{k-1,{\textbf{z}}}\right) _{{\text {mvz}}} \in {M_{\eta ,k-1} \left( {\mathcal {T}} \right) }^{\perp }$ for all ${\textbf{z}}\in {{\mathcal {S}}}{{\mathcal {C}}}_{{\mathcal {T}}}(\eta )$ from Lemma 4.7 provide ${\text {dim}} M_{\eta ,k-1} \left( {\mathcal {T}} \right) = {\text {dim}} M_{\eta ,k-1}^{{\text {mod}}} \left( {\mathcal {T}} \right)$. Hence, ${\text {card}} {\mathcal {B}} = {\text {dim}} M_{\eta ,k-1}^{{\text {mod}}} \left( {\mathcal {T}} \right) ^{\perp }$. The set $\left\{ \left. b_{k-1,{\textbf{z}}}\;\right| \;\forall {\textbf{z}}\in {\mathcal {C}} _{{\mathcal {T}}}\left( \eta \right) {\setminus }{{\mathcal {S}}}{{\mathcal {C}}}_{{\mathcal {T}}}\left( \eta \right) \right\}$ is linearly independent by Proposition 3.4 and orthogonal to both, $M_{\eta ,k-1}^{{\text {mod}}}\left( {\mathcal {T}}\right) \subset M_{\eta ,k-1}({\mathcal {T}})+\{\left( b_{k-1,{\textbf{z}}}\right) _{{\text {mvz}}}\ |\ {\textbf{z}}\in {\mathcal {S}}{\mathcal {C}}_{{\mathcal {T}}}(\eta )\}$ and ${\mathcal {B}}_0{:}{=}\{\varphi _{{\textbf{z}}}\ |\ \forall {\textbf{z}}\in {\mathcal {S}}{\mathcal {C}}_{{\mathcal {T}}}(\eta )\}\subset {\mathcal {B}}$, by Lemma 4.7. To analyse the remaining subset ${\mathcal {B}}_0$, let

$$\begin{aligned} q=q_{0}+\sum _{{\textbf{y}}\in {{\mathcal {S}}}{{\mathcal {C}}}_{{\mathcal {T}}}\left( \eta \right) }f_{{\textbf{y}}}\left( q_{0}\right) \left( b_{k-1,{\textbf{y}}}\right) _{{\text {mvz}}}\in M_{\eta ,k-1}^{{\text {mod}}} \left( {\mathcal {T}} \right) \end{aligned}$$

be arbitrary with $q_{0}\in M_{\eta ,k-1}\left( {\mathcal {T}}\right)$. Given any ${\textbf{z}}\in {{\mathcal {S}}}{{\mathcal {C}}}_{{\mathcal {T}}}\left( \eta \right)$, consider

4.13 $$\begin{aligned} \left( \varphi _{{\textbf{z}}},q\right) _{L^{2}\left( \Omega \right) }=\left( \varphi _{{\textbf{z}}},q_{0}\right) _{L^{2}\left( \Omega \right) } +\sum _{{\textbf{y}}\in {{\mathcal {S}}}{{\mathcal {C}}}_{{\mathcal {T}}}\left( \eta \right) }f_{{\textbf{y}}}\left( q_{0}\right) \left( \varphi _{{\textbf{z}}},\left( b_{k-1,{\textbf{y}}}\right) _{{\text {mvz}}}\right) _{L^{2}\left( \Omega \right) }. \end{aligned}$$

The definition of $\varphi _{{\textbf{z}}}$ in (4.12) with (4.11) and $\left( \left( b_{{\textbf{z}},k-1}\right) _{{\text {mvz}}},q_{0}\right) _{L^{2}\left( \Omega \right) }=0$ by Lemma 4.7 reveal

4.14 $$\begin{aligned} \left( \varphi _{{\textbf{z}}},q_{0}\right) _{L^{2}\left( \Omega \right) }&=f_{{\textbf{z}}}\left( q_{0}\right) -\frac{1}{\left\| b_{k-1,{\textbf{z}} }\right\| _{L^{2}\left( \Omega \right) }^{2}}\sum _{{\textbf{y}} \in {{\mathcal {S}}}{{\mathcal {C}}}_{{\mathcal {T}}}\left( \eta \right) }\left( \overline{b_{k-1,{\textbf{z}}}},b_{k-1,{\textbf{y}}}\right) _{L^{2}\left( \Omega \right) }f_{{\textbf{y}}}\left( q_{0}\right) . \end{aligned}$$

A similar computation with $\left( \phi _{{\textbf{a}}},\left( b_{k-1,{\textbf{y}}}\right) _{{\text {mvz}}}\right) _{L^{2}\left( \Omega \right) } = 0$ for all ${\textbf{a}}\in {\mathcal {S}}{\mathcal {C}}_{{\mathcal {T}}}(\eta )$ from $\phi _{{\textbf{z}}}\in M_{\eta ,k-1}\left( {\mathcal {T}}\right)$ and Lemma 4.7 implies for all ${\textbf{z}}\in {\mathcal {S}}{\mathcal {C}}_{{\mathcal {T}}}(\eta )$ that

$$\begin{aligned} \left( \varphi _{{\textbf{z}}},\left( b_{k-1,{\textbf{y}}}\right) _{{\text {mvz}}}\right) _{L^{2}\left( \Omega \right) }&=-\frac{\left( \left( b_{k-1,{\textbf{z}}}\right) _{{\text {mvz}}},\left( b_{k-1,{\textbf{y}} }\right) _{{\text {mvz}}}\right) _{L^{2}\left( \Omega \right) } }{\left\| b_{k-1,{\textbf{z}}}\right\| _{L^{2}\left( \Omega \right) } ^{2}} \\&= -\delta _{{\textbf{z}},{\textbf{y}}}+\frac{\left( \overline{b_{k-1,{\textbf{z}} }},b_{k-1,{\textbf{y}}}\right) _{L^{2}\left( \Omega \right) }}{\left\| b_{k-1,{\textbf{z}}}\right\| _{L^{2}\left( \Omega \right) }^{2}} \end{aligned}$$

using the integral mean zero property of $\left( b_{k-1,{\textbf{z}}}\right) _{{\text {mvz}}}$ and Remark 3.12(i) as all super-critical vertices are of Robinson type in the last step. Here, $\delta _{{\textbf{z}},{\textbf{y}}}$ denotes the Kronecker delta. The sum over all ${\textbf{y}}\in {\mathcal {S}}{\mathcal {C}}_{{\mathcal {T}}}(\eta )$ results in

4.15 $$\begin{aligned} \sum _{{{\textbf {y}}}\in {{\mathcal {S}}}{{\mathcal {C}}}_{{\mathcal {T}}}\left( \eta \right) }f_{{{\textbf {y}}}}&\left( q_{0}\right) \left( \varphi _{{{\textbf {z}}}},\left( b_{k-1,{{\textbf {y}}}}\right) _{{\text {mvz}}}\right) _{L^{2}\left( \Omega \right) } \nonumber \\  &=-f_{{{\textbf {z}}}}\left( q_{0}\right) +\sum _{{{\textbf {y}}}\in {{\mathcal {S}}}{{\mathcal {C}}} _{{\mathcal {T}}}\left( \eta \right) }f_{{{\textbf {y}}}}\left( q_{0}\right) \frac{\left( \overline{b_{k-1,{{\textbf {z}}}}},b_{k-1,{{\textbf {y}}}}\right) _{L^{2}\left( \Omega \right) }}{\left\| b_{k-1,{{\textbf {z}}}}\right\| _{L^{2}\left( \Omega \right) }^{2}}. \end{aligned}$$

The conclusion of (4.13)–(4.15) reads $\left( \varphi _{{\textbf{z}}},q\right) _{L^{2}\left( \Omega \right) }=0$ and, since ${\textbf{z}}\in {\mathcal {S}}{\mathcal {C}}_{{\mathcal {T}}}(\eta )$ was arbitrary, ${\mathcal {B}}_0\subseteq M_{\eta ,k-1}^{{\text {mod}} }\left( {\mathcal {T}}\right) ^{\perp }$. It remains to show that ${\mathcal {B}}_0$ is linearly independent. Given any $c_{{\textbf{z}}}\in {\mathbb {R}}$ for ${\textbf{z}}\in {\mathcal {S}}{\mathcal {C}}_{{\mathcal {T}}}(\eta )$ with $\sum _{{\textbf{z}}\in {{\mathcal {S}}}{{\mathcal {C}}}_{{\mathcal {T}}}\left( \eta \right) }c_{{\textbf{z}}}\varphi _{{\textbf{z}}}=0$, the definition of $\varphi _{{\textbf{z}}}$ in (4.12) reveals

$$\begin{aligned} 0&=\sum _{{\textbf{z}}\in {{\mathcal {S}}}{{\mathcal {C}}}_{{\mathcal {T}}}\left( \eta \right) }\left( C_{{\textbf{z}}}\phi _{{\textbf{z}}}-c_{{\textbf{z}}}\frac{\left( b_{k-1,{\textbf{z}} }\right) _{{\text {mvz}}}}{\left\| b_{k-1,{\textbf{z}} }\right\| _{L^{2}\left( \Omega \right) }^{2}}\right) \end{aligned}$$

with $C_{{\textbf{z}}}{:}{=}c_{{\textbf{z}}}-\sum  _{{\textbf{z}}\in {\mathcal {S}}{\mathcal {C}}_{{\mathcal {T}}}(\eta )}\left( \overline{b_{k-1,{\textbf{z}}}},b_{k-1,{\textbf{y}}}\right) _{L^{2}\left( \Omega \right) }/\left\| b_{k-1,{\textbf{z}} }\right\| _{L^{2}\left( \Omega \right) }^{2}$. This leads to the condition

$$\begin{aligned} T:=\sum _{{\textbf{z}}\in {{\mathcal {S}}}{{\mathcal {C}}}_{{\mathcal {T}}}\left( \eta \right) } \frac{c_{{\textbf{z}}}}{\left\| b_{k-1,{\textbf{z}}}\right\| _{L^{2}\left( \Omega \right) }^{2}}\left( b_{k-1,{\textbf{z}}}\right) _{{\text {mvz}} }=\sum _{{\textbf{y}}\in {{\mathcal {S}}}{{\mathcal {C}}}_{{\mathcal {T}}}\left( \eta \right) }C_{{\textbf{y}}}\phi _{{\textbf{y}}}\in M_{\eta ,k-1}({\mathcal {T}}). \end{aligned}$$

Hence, $T\in {\text {span}}\{\left( b_{k-1,{\textbf{z}}}\right) _{{\text {mvz}} }\ |\ {\textbf{z}}\in {\mathcal {S}}{\mathcal {C}}_{{\mathcal {T}}}(\eta )\}\cap M_{\eta ,k-1}({\mathcal {T}})=\{0\}$ vanishes by Lemma 4.7. Since the $\left( b_{k-1,{\textbf{z}}}\right) _{{\text {mvz}}}$ are linear independent by Proposition 3.4, $T=0$ implies $c_{{\textbf{z}}}=0$ for all ${\textbf{z}}\in {\mathcal {S}}{\mathcal {C}}_{{\mathcal {T}}}(\eta )$. This verifies the linear independence of ${\mathcal {B}}_0$ and concludes the proof. $\square$

The following lemma recalls the key estimates for the divergence control in [13].

#### Proposition 4.12

([13])

- Given $0\le \eta \le 1$, any $q\in M_{0,k-1}({\mathcal {T}})\cap M_{\eta ,k-1}({\mathcal {T}})^\perp$ satisfies
  $$\begin{aligned} \left\| q\right\| _{L^{2}\left( \Omega \right) }^2\le \frac{12}{7}\left( {\begin{array}{c}k+1\\ 2\end{array}}\right) ^{-2}\sum _{{\textbf{z}}\in {\mathcal {C}}_{{\mathcal {T}}}\left( \eta \right) }h_{{\textbf{z}} }^{2}\left( A_{{\mathcal {T}},{\textbf{z}}}\left( q\right) \right) ^{2}. \end{aligned}$$
- There exists $\eta _0>0$ exclusively depending on the shape-regularity of the mesh and the minimal outer angle such that for any $0\le \eta <\eta _0$ and any ${\textbf{z}}\in {\mathcal {C}}_{{\mathcal {T}}}(\eta )$ we have
  $$\begin{aligned} \left| A_{{\mathcal {T}} ,{\textbf{z}}}\left( {\text {div}}{\textbf{v}}\right) \right| \le Ch_{{\textbf{z}}} ^{-1}k^{2}\eta \left\| \nabla {\textbf{v}}\right\| _{{\mathbb {L}}^{2}\left( \omega _{{\textbf{z}}}\right) }\quad \forall {\textbf{v}}\in {\textbf{S}} _{k,0}\left( {\mathcal {T}}\right) . \end{aligned}$$
  The constant $C>0$ depends only on the shape-regularity of the mesh.

#### Proof

The estimate in (a) is the conclusion of Step 2 of the proof of Lemma 4 in [13], therein stated for the divergence ${\text {div}}{\textbf{v}}_{{\textbf{S}}}=q$ of the right-inverse ${\textbf{v}}_{{\textbf{S}}}=\Pi _kq\in {\textbf{S}}_{k,0}({\mathcal {T}})$ from Proposition 3.8. The estimate in (b) is provided in [13, Cor. 1 on p. 16] in terms of $\eta _0$ from [19, Lem. 2.13] discussed in Remark 4.2; further details are omitted. $\square$

#### Proof of Theorem 4.10

The proof of Theorem 4.10 consists of three steps.

**Step 1: preparations.** Since ${\text {div}}{\textbf{u}}_{{\textbf{S}}}\in M_{\eta ,k-1}^{{\text {mod}}}({\mathcal {T}})^\perp$ by (4.10), Lemma 4.11 provides

4.16 $$\begin{aligned} {\text {div}}{\textbf{u}}_{{\textbf{S}}}=\sum _{{\textbf{z}}\in {\mathcal {C}} _{{\mathcal {T}}}\left( \eta \right) \setminus {{\mathcal {S}}}{{\mathcal {C}}}_{{\mathcal {T}}}\left( \eta \right) }c_{{\textbf{z}}}b_{k-1,{\textbf{z}}}+\sum _{{\textbf{y}}\in {{\mathcal {S}}}{{\mathcal {C}}}_{{\mathcal {T}}}\left( \eta \right) }c_{{\textbf{y}}}\varphi _{{\textbf{y}}}=q_{1}+q_{2}+q_{3}, \end{aligned}$$

for coefficients $c_{{\textbf{z}}},c_{{\textbf{y}}}, C_{{\textbf{y}}}\in {\mathbb {R}}$ and

$$\begin{aligned}&q_{1} :=\hspace{-.7em}\sum _{{\textbf{z}}\in {\mathcal {C}}_{{\mathcal {T}}}\left( \eta \right) \setminus {{\mathcal {S}}}{{\mathcal {C}}}_{{\mathcal {T}}}\left( \eta \right) }\hspace{-1em}c_{{\textbf{z}} }b_{k-1,{\textbf{z}}},\\&q_{2}:=-\hspace{-0.5em}\sum _{{\textbf{y}}\in {{\mathcal {S}}}{{\mathcal {C}}}_{{\mathcal {T}}}\left( \eta \right) }\frac{c_{{\textbf{y}}}}{\left\| b_{k-1,{\textbf{y}}}\right\| _{L^{2}\left( \Omega \right) }^{2}}\left( b_{k-1,{\textbf{y}}}\right) _{{\text {mvz}} },\\&q_{3} :=\hspace{-.3em}\sum _{{\textbf{y}}\in {{\mathcal {S}}}{{\mathcal {C}}}_{{\mathcal {T}}}\left( \eta \right) }C_{{\textbf{y}}} \phi _{{\textbf{y}}}. \end{aligned}$$

More precisely, the coefficients $C_{{\textbf{y}}}$ are given for all ${\textbf{y}}\in {\mathcal {S}}{\mathcal {C}}_{{\mathcal {T}}}(\eta )$ in terms of $c_{{\textbf{y}}}$ as

4.17 $$\begin{aligned} C_{{\textbf{y}}}&:=c_{{\textbf{y}}}-\sum _{{\textbf{a}}\in {{\mathcal {S}}}{{\mathcal {C}}}_{{\mathcal {T}}}\left( \eta \right) }c_{{\textbf{a}}}\frac{\left( \overline{b_{k-1,{\textbf{a}}} },b_{k-1,{\textbf{y}}}\right) _{L^{2}\left( \Omega \right) }}{\left\| b_{k-1,{\textbf{a}}}\right\| _{L^{2}\left( \Omega \right) }^{2}}. \end{aligned}$$

Since ${{\mathcal {S}}}{{\mathcal {C}}}_{{\mathcal {T}}}\left( \eta \right)$ contains only Robinson vertices by assumption, the functions $q_{1}$, $q_{2}$, $q_{3}$ are pairwise orthogonal by Lemma 4.7 and $\phi _{{\textbf{z}}}\in M_{\eta ,k-1}\left( {\mathcal {T}}\right)$ for all ${\textbf{z}}\in {\mathcal {C}}_{{\mathcal {T}}}\left( \eta \right) {\setminus }{{\mathcal {S}}}{{\mathcal {C}}}_{{\mathcal {T}}}\left( \eta \right)$. Recall ${\text {div}} {\textbf{u}}_{{\textbf{S}}}\in M_{0,k-1}\left( {\mathcal {T}}\right)$ from (3.11) so that $q_{1},q_{2}\in M_{\eta ,k-1}\left( {\mathcal {T}}\right) ^{\perp }$ and $q_{3}\in M_{\eta ,k-1}\left( {\mathcal {T}}\right) \subseteq M_{0,k-1}\left( {\mathcal {T}}\right)$ imply

4.18 $$\begin{aligned} {\text {div}}{\textbf{u}}_{{\textbf{S}}}-q_{3}=q_1+q_2 \in M_{0,k-1}\left( {\mathcal {T}}\right) \cap M_{\eta ,k-1}\left( {\mathcal {T}}\right) ^{\perp }. \end{aligned}$$

**Step 2: control of **$q_{3}$**.** By assumption, (3.27)–(3.28) hold for all ${\textbf{z}}\in {\mathcal {S}}{\mathcal {C}}_{{\mathcal {T}}}(\eta )$. In particular, (3.28) bounds the operator norm $\left\| f_{{\textbf{z}}}\right\| _{{\mathbb {R}}\leftarrow M_{\eta ,k-1}}$ of $f_{{\textbf{z}}}$ and reveals with (4.11) that

$$\begin{aligned} \left\| \phi _{{\textbf{z}} }\right\| _{L^{2}\left( \Omega \right) }=\left\| f_{{\textbf{z}} }\right\| _{{\mathbb {R}}\leftarrow M_{\eta ,k-1}}\le C_{f_{{\textbf{z}}} }/\left\| b_{k-1,{\textbf{z}}}\right\| _{L^{2}\left( \Omega \right) }. \end{aligned}$$

This, triangle and Cauchy-Schwarz inequalities (in $\ell ^2$) result for ${\widetilde{C}}_{f}:=\sqrt{\sum  _{{\textbf{y}}\in {\mathcal {S}}{\mathcal {C}}_{{\mathcal {T}}}(\eta )} C_{f_{{\textbf{y}}}}^2}$ in

4.19 $$\begin{aligned} \left\| q_{3}\right\| _{L^{2}\left( \Omega \right) }&\le \sum  _{{\textbf{y}}\in {\mathcal {S}}{\mathcal {C}}_{{\mathcal {T}}}(\eta )} \frac{C_{{\textbf{y}}}}{\Vert b_{k-1,{\textbf{y}}}\Vert _{L^2(\Omega )}}C_{f_{{\textbf{y}}}} \le {\widetilde{C}}_f \sqrt{\sum  _{{\textbf{y}}\in {\mathcal {S}}{\mathcal {C}}_{{\mathcal {T}}}(\eta )} \frac{C_{{\textbf{y}}}^2}{\Vert b_{k-1,{\textbf{y}}}\Vert _{L^2(\Omega )}^2}}. \end{aligned}$$

The orthogonality $\left\| \left( b_{k-1,{\textbf{y}}}\right) _{{\text {mvz}}}\right\| _{L ^{2}(\Omega )}^2+\left\| \overline{b_{k-1,{\textbf{z}}}}\right\| _{L ^{2}\left( \Omega \right) }^2 = \left\| {b_{k-1,{\textbf{z}}}}\right\| _{L ^{2}\left( \Omega \right) }^2$ of the $L^{2}$-projection, the Cauchy-Schwarz inequality, and the definition of $C_{{\textbf{y}}}$ in (4.17) reveal

$$\begin{aligned} C_{{\textbf{y}}}\le c_{{\textbf{y}}}\frac{\Vert \left( b_{k-1,{\textbf{y}}}\right) _{{\text {mvz}} }\Vert _{L^2(\Omega )}}{\left\| b_{k-1,{\textbf{y}}}\right\| _{L^{2}\left( \Omega \right) } }+\sum _{{\textbf{a}}\in {{\mathcal {S}}}{{\mathcal {C}}}_{{\mathcal {T}}}\left( \eta \right) \setminus \{{\textbf{y}}\} }c_{{\textbf{a}}}\frac{\Vert {b_{k-1,{\textbf{y}}} }\Vert _{L^2(\Omega )}}{\left\| b_{k-1,{\textbf{a}}}\right\| _{L \left( \Omega \right) }^{2}} \le \sum _{{\textbf{a}}\in {{\mathcal {S}}}{{\mathcal {C}}}_{{\mathcal {T}}}\left( \eta \right) }c_{{\textbf{a}}}\frac{\left\| b_{k-1,{\textbf{y}}}\right\| _{L^{2}\left( \Omega \right) }}{\left\| b_{k-1,{\textbf{a}}}\right\| _{L^{2}\left( \Omega \right) }}. \end{aligned}$$

The previous two estimates and a Cauchy-Schwarz inequality imply for $C_{0}={\widetilde{C}}_f^2{\text {card}}{{\mathcal {S}}}{{\mathcal {C}}}_{{\mathcal {T}}}\left( \eta \right) ^2$ that

4.20 $$\begin{aligned} \left\| q_{3}\right\| _{L^{2}\left( \Omega \right) }^{2} \le {\widetilde{C}}_f^2 \sum  _{{\textbf{y}}\in {\mathcal {S}}{\mathcal {C}}_{{\mathcal {T}}}(\eta )} \left( \sum  _{{\textbf{a}}\in {\mathcal {S}}{\mathcal {C}}_{{\mathcal {T}}}(\eta )} \frac{c_{{\textbf{a}}}}{\Vert b_{k-1,{\textbf{a}}}\Vert _{L^2(\Omega )}}\right) ^2 \le C_{0} ^{2}\sum _{{\textbf{y}}\in {{\mathcal {S}}}{{\mathcal {C}}}_{{\mathcal {T}}}\left( \eta \right) } \frac{c_{{\textbf{y}}}^{2}}{\left\| b_{k-1,{\textbf{y}}}\right\| _{L^{2}\left( \Omega \right) }^{2}}. \end{aligned}$$

All $\eta$-super critical vertices are of Robinson type by assumption and $\big \{ b_{k-1,{\textbf{y}}}\;\big \vert \;{\textbf{y}}\in {{\mathcal {S}}}{{\mathcal {C}}}_{{\mathcal {T}}}\left( \eta \right) \big \}$ is pairwise orthogonal by Remark 3.12.(ii). Hence, the sum in the right-hand side of (4.20) is the $L^2$ norm of

$$\begin{aligned} {\tilde{q}}_{2}:=-\sum _{{\textbf{y}}\in {{\mathcal {S}}}{{\mathcal {C}}}_{{\mathcal {T}}}\left( \eta \right) }\frac{c_{{\textbf{y}}}}{\left\| b_{k-1,{\textbf{y}}}\right\| _{L^{2}\left( \Omega \right) }^2}b_{k-1,{\textbf{y}}}. \end{aligned}$$

A comparison with the definition of $q_2$ verifies $q_{2}=\left( {\tilde{q}}_{2}\right) _{{\text {mvz}}}$ and (3.17) in Proposition 3.4 shows

$$\begin{aligned} C_{1}^{-2}\Vert q_3\Vert _{L^2(\Omega )}^2\le \left\| {\tilde{q}}_{2}\right\| _{L^{2}\left( \Omega \right) }^{2} \le \frac{16}{7}\sum _{K\in {\mathcal {T}}}\inf _{\alpha \in {\mathbb {R}}}\left\| {\tilde{q}}_{2}-\alpha \right\| _{L^{2}\left( K\right) }^{2}\le \frac{16}{7}\left\| q_{2}\right\| _{L^{2}\left( \Omega \right) }^{2}. \end{aligned}$$

The conclusion of the previous estimates is the existence of $C>0$ exclusively depending on the cardinality of ${{\mathcal {S}}}{{\mathcal {C}}}_{{\mathcal {T}}}\left( \eta \right)$ and the constants $C_{f_{{\textbf{y}}}}$ such that

4.21 $$\begin{aligned} \left\| q_{3}\right\| _{L^{2}\left( \Omega \right) }\le C\left\| q_{2}\right\| _{L^{2}\left( \Omega \right) }. \end{aligned}$$

**Step 3: Conclusion.** The pairwise orthogonality of $q_1,q_2,q_3$ with ${\text {div}}{\textbf{u}}_{{\textbf{S}}}=q_1+q_2+q_3$ from Step 1 and (4.21) verify

4.22 $$\begin{aligned} \left\| {\text {div}}{\textbf{u}}_{{\textbf{S}}}\right\| _{L^{2}\left( \Omega \right) }^{2}&=\left\| q_{1}\right\| _{L^{2}\left( \Omega \right) }^{2}+\left\| q_{2}\right\| _{L^{2}\left( \Omega \right) }^{2}+\left\| q_{3}\right\| _{L^{2}\left( \Omega \right) }^{2} \le \left\| q_{1}\right\| _{L^{2}\left( \Omega \right) }^{2}+\left( 1+C\right) \left\| q_{2}\right\| _{L^{2}\left( \Omega \right) } ^{2}\nonumber \\&\le \left( 1+C\right) \left\| q_{1}+q_{2}\right\| _{L^{2}\left( \Omega \right) }^{2}. \end{aligned}$$

Proposition 4.12.(a) with (4.18) and $A_{{\mathcal {T}},{\textbf{z}}}(q_3)=0$ from $q_3\in M_{\eta ,k-1}({\mathcal {T}})$ provide

4.23 $$\begin{aligned} \left\| q_{1}+q_{2}\right\| _{L^{2}\left( \Omega \right) }^{2} \le C\sum _{{\textbf{z}}\in {\mathcal {C}}_{{\mathcal {T}}}\left( \eta \right) }h_{{\textbf{z}}}^{2}\left[ A_{{\mathcal {T}},{\textbf{z}}}\left( q_{1} +q_{2}\right) \right] ^{2} \le C\sum _{{\textbf{z}}\in {\mathcal {C}}_{{\mathcal {T}}}\left( \eta \right) }h_{{\textbf{z}}}^{2}\left[ A_{{\mathcal {T}},{\textbf{z}}}\left( {\text {div}}{\textbf{u}}_{{\textbf{S}}}\right) \right] ^{2}. \end{aligned}$$

By definition of ${\textbf{S}}_{\eta ,k,0} \left( {\mathcal {T}}\right)$ in (4.8), $A_{{\mathcal {T}},{\textbf{y}}}({\text {div}}{\textbf{w}}_{{\textbf{S}}}) =0$ vanishes for all ${\textbf{w}}_{{\textbf{S}}}\in {\textbf{S}}_{\eta ,k,0} \left( {\mathcal {T}}\right)$ and ${\textbf{y}}\in {\mathcal {C}}_{{\mathcal {T}}}(\eta )$. Proposition 4.12(b) provides $0<\eta _0$ such that this and the combination of (4.22)–(4.23) reveal for $0\le \eta < \eta _0$ that

$$\begin{aligned} \left\| {\text {div}}{\textbf{u}}_{{\textbf{S}}}\right\| _{L^{2}\left( \Omega \right) }^{2}\le C\sum _{{\textbf{z}}\in {\mathcal {C}}_{{\mathcal {T}}}\left( \eta \right) }h_{{\textbf{z}}}^{2}A_{{\mathcal {T}},{\textbf{z}}}\left( {\text {div}}\left( {\textbf{u}}_{{\textbf{S}}}-{\textbf{w}}_{{\textbf{S}} }\right) \right) ^{2}\le C\eta ^{2}\left\| \nabla \left( {\textbf{u}} _{{\textbf{S}}}-{\textbf{w}}_{{\textbf{S}}}\right) \right\| _{{\mathbb {L}} ^{2}\left( \Omega (\eta )\right) }^{2} \end{aligned}$$

using the finite overlay of the vertex patches $\{\omega _{{\textbf{z}}}\ |\ {\textbf{z}}\in {\mathcal {C}}_{{\mathcal {T}}}(\eta )\}$ in the neighbourhood $\Omega (\eta )$ from (4.7). This concludes the proof with $\eta _0$ from Proposition 4.12(b). $\square$

With this we obtain an analogous estimates as in [13, Thm. 3].

#### Proposition 4.13

Under the Assumptions of Theorem 4.10 with the solution $\left( {\textbf{u}},p\right)$ to (2.2) for ${\textbf{F}}\in {\textbf{H}}^{-1}\left( \Omega \right)$, the discrete solution $\left( {\textbf{u}}_{{\textbf{S}}},p_{M}^{*}\right) \in \left( {\textbf{S}}_{k,0}\left( {\mathcal {T}}\right) ,M_{\eta ,k-1}^{{\text {mod}}}\left( {\mathcal {T}}\right) \right)$ to (2.3) satisfies

4.24a $$\begin{aligned} \left\| \nabla \left( {\textbf{u}}-{\textbf{u}}_{{\textbf{S}}}\right) \right\| _{{\mathbb {L}}^{2}\left( \Omega \right) }&\le C\left( \inf _{\begin{array}{c} {\textbf{v}}_{{\textbf{S}}}\in {\textbf{S}}_{k,0}\left( {\mathcal {T}}\right) \\ {\text {div}}{\textbf{v}}_{{\textbf{S}}}=0 \end{array}}\left\| \nabla \left( {\textbf{u}}-{\textbf{v}}_{{\textbf{S}}}\right) \right\| _{{\mathbb {L}}^{2}\left( \Omega \right) }+\eta \inf _{q_{M}\in M_{\eta ,k-1}^{{\text {mod}}}\left( {\mathcal {T}}\right) }\left\| p-q_{M}\right\| _{L^{2}\left( \Omega \right) }\right) , \end{aligned}$$

4.24b $$\begin{aligned} \left\| {\text {div}}{\textbf{u}}_{{\textbf{S}}}\right\| _{L^{2}\left( \Omega \right) }&\le C\left( \eta \inf _{\begin{array}{c} {\textbf{v}}_{{\textbf{S}} }\in {\textbf{S}}_{k,0}\left( {\mathcal {T}}\right) \\ {\text {div}} {\textbf{v}}_{{\textbf{S}}}=0 \end{array}}\left\| \nabla \left( {\textbf{u}}-{\textbf{v}} _{{\textbf{S}}}\right) \right\| _{{\mathbb {L}}^{2}\left( \Omega \right) }+\eta ^{2}\inf _{q_{M}\in M_{\eta ,k-1}^{{\text {mod}}}\left( {\mathcal {T}}\right) }\left\| p-q_{M}\right\| _{L^{2}\left( \Omega \right) }\right) . \end{aligned}$$

#### Proof

We start with the first estimate (4.24a) and follow the arguments of the proof of [13, Thm. 3]. Since the discrete velocity ${\textbf{u}}_{{\textbf{S}}}$ satisfies the second equation in (2.3) we conclude (4.10). Next, consider some ${\textbf{v}}_{{\textbf{S}}}\in {\textbf{S}}_{k,0}\left( {\mathcal {T}}\right)$ with ${\text {div}}{\textbf{v}}_{{\textbf{S}}}=0$ and observe that ${\textbf{v}}_{{\textbf{S}}}\in {\textbf{S}}_{\eta ,k,0}\left( {\mathcal {T}}\right)$, such that the first equations in (2.2) and (2.3) for the test function ${\textbf{e}}_{{\textbf{S}}}={\textbf{u}}_{{\textbf{S}}}-{\textbf{v}}_{{\textbf{S}}}$ verifies

$$\begin{aligned} a\left( {\textbf{u}}-{\textbf{u}}_{{\textbf{S}}},{\textbf{e}}_{{\textbf{S}}}\right) =b\left( {\textbf{e}}_{{\textbf{S}}},p-p_{M}\right) . \end{aligned}$$

Since ${\text {div}}{\textbf{e}}_{{\textbf{S}}}={\text {div}} {\textbf{u}}_{{\textbf{S}}}$ is orthogonal to $M_{\eta ,k-1}^{{\text {mod}} }\left( {\mathcal {T}}\right)$ we get

$$\begin{aligned} a\left( {\textbf{u}}-{\textbf{u}}_{{\textbf{S}}},{\textbf{e}}_{{\textbf{S}}}\right) =b\left( {\textbf{u}}_{{\textbf{S}}},p-q_{M}\right) \qquad \forall q_{M}\in M_{\eta ,k-1}^{{\text {mod}}}\left( {\mathcal {T}}\right) . \end{aligned}$$

The combination of these relations leads for any $q_{M}\in M_{\eta ,k-1}^{{\text {mod}}}\left( {\mathcal {T}}\right)$ to

$$\begin{aligned} \left\| \nabla \left( {\textbf{u}}_{{\textbf{S}}}-{\textbf{v}}_{{\textbf{S}} }\right) \right\| _{{\mathbb {L}}^{2}\left( \Omega \right) }^{2}&=a\left( {\textbf{u}}_{{\textbf{S}}}-{\textbf{v}}_{{\textbf{S}}},{\textbf{e}} _{{\textbf{S}}}\right) =a\left( {\textbf{u}}-{\textbf{v}}_{{\textbf{S}}} ,{\textbf{e}}_{{\textbf{S}}}\right) -a\left( {\textbf{u}}-{\textbf{u}}_{{\textbf{S}} },{\textbf{e}}_{{\textbf{S}}}\right) \\&\le \left\| \nabla \left( {\textbf{u}}-{\textbf{v}}_{{\textbf{S}}}\right) \right\| _{{\mathbb {L}}^{2}\left( \Omega \right) }\left\| \nabla {\textbf{e}}_{{\textbf{S}}}\right\| _{{\mathbb {L}}^{2}\left( \Omega \right) }+\left\| {\text {div}}{\textbf{u}}_{{\textbf{S}}}\right\| _{L^{2}\left( \Omega \right) }\left\| p-q_{M}\right\| _{L^{2}\left( \Omega \right) }. \end{aligned}$$

From (4.9) we obtain for the choice ${\textbf{w}}_{{\textbf{S}} }={\textbf{v}}_{{\textbf{S}}}$, that

4.25 $$\begin{aligned} \left\| {\text {div}}{\textbf{u}}_{{\textbf{S}}}\right\| _{L^{2}\left( \Omega \right) }\le C_{{\text {div}}}\eta \left\| \nabla \left( {\textbf{u}}_{{\textbf{S}}}-{\textbf{v}}_{{\textbf{S}}}\right) \right\| _{\mathbb {L}^{2}\left( \Omega \right) } \end{aligned}$$

and, in turn,

$$\begin{aligned} \left\| \nabla \left( {\textbf{u}}_{{\textbf{S}}}-{\textbf{v}}_{{\textbf{S}} }\right) \right\| _{{\mathbb {L}}^{2}\left( \Omega \right) }\le \left\| \nabla \left( {\textbf{u}}-{\textbf{v}}_{{\textbf{S}}}\right) \right\| _{{\mathbb {L}}^{2}\left( \Omega \right) }+C_{{\text {div}}}\eta \left\| p-q_{M}\right\| _{L^{2}\left( \Omega \right) }. \end{aligned}$$

Thus, the velocity error can be estimated by

$$\begin{aligned} \left\| \nabla \left( {\textbf{u}}-{\textbf{u}}_{{\textbf{S}}}\right) \right\| _{{\mathbb {L}}^{2}\left( \Omega \right) }&\le \left\| \nabla \left( {\textbf{u}}-{\textbf{v}}_{{\textbf{S}}}\right) \right\| _{{\mathbb {L}}^{2}\left( \Omega \right) }+\left\| \nabla \left( {\textbf{u}}_{{\textbf{S}}}-{\textbf{v}}_{{\textbf{S}}}\right) \right\| _{{\mathbb {L}}^{2}\left( \Omega \right) }\nonumber \\&\le 2\left\| \nabla \left( {\textbf{u}}-{\textbf{v}}_{{\textbf{S}}}\right) \right\| _{{\mathbb {L}}^{2}\left( \Omega \right) }+C_{{\text {div}} }\eta \inf _{q_{M}\in M_{\eta ,k-1}^{{\text {mod}}}\left( {\mathcal {T}} \right) }\left\| p-q_{M}\right\| _{L^{2}\left( \Omega \right) }. \end{aligned}$$

Since ${\textbf{v}}_{{\textbf{S}}}$ was chosen arbitrarily, we obtain (4.24a) for $C = 2 + C_{{\text {div}}}$. For (4.24b), we take (4.25) and obtain through a triangle inequality that

$$\begin{aligned} \left\| {\text {div}}{{\textbf {u}}}_{{{\textbf {S}}}}\right\| _{L^{2}\left( \Omega \right) }&\le C_{{\text {div}}} \eta \left( \left\| \nabla \left( {{\textbf {u}}} - {{\textbf {u}}}_{{{\textbf {S}}}} \right) \right\| _{\mathbb L ^{2}\left( \Omega \right) } + \left\| \nabla ({{\textbf {u}}} - {{\textbf {v}}}_{{{\textbf {S}}}}) \right\| _{\mathbb {L} ^{2}\left( \Omega \right) }\right) . \end{aligned}$$

This and (4.24a) reveals (4.24b) concluding the proof. $\square$

Two options for obtaining convergence rates for the discrete solution from the quasi-optimal error estimates are sketched in the following remark.

#### Remark 4.14

Let $\left( {\textbf{u}}_{{\textbf{S}}},p_{M}^{*}\right) \in \left( {\textbf{S}}_{k,0}\left( {\mathcal {T}}\right) ,M_{\eta ,k-1} ^{{\text {mod}}}\left( {\mathcal {T}}\right) \right)$ denote the discrete solution of the Stokes problem.

**1. **The combination of Corollary 4.6 with Theorem 4.9 directly results in *h*-explicit convergence rates for the infima in the quasi-optimal error estimates (4.4). A minor drawback is that in this way the pressure infima are not multiplied by the small pre-factor $\eta$ in (4.4) in contrast to (4.24), i.e., the resulting estimate is less *pressure-robust.*

**2. **A direct estimate of the best-approximation of the velocity in (4.24) can be obtained by selecting for ${\textbf{v}}_{{\textbf{S}}}$ the original (solenoidal) Scott–Vogelius velocity field ${\textbf{v}} _{{\textbf{S}}}^{{\text {SV}}}$. However, the stability bound of ${\textbf{v}}_{{\textbf{S}}}^{{\text {SV}}}$ is reciprocally related to discrete inf-sup constant of the original Scott–Vogelius element and may become large if the mesh has nearly-singular vertices.

A direct construction of some *solenoidal* ${\textbf{v}}_{{\textbf{S}}} \in {\textbf{S}}_{k,0}\left( {\mathcal {T}}\right)$ which leads to optimal, mesh-robust convergence rates for the first infima in (4.24) is, to the best of our knowledge, an open question.

## A Approximation by polynomial extension

The proofs for the approximation property of our modified pressure spaces rely on some estimates for the analytic extension of polynomials (extension by “itself”) proved in this appendix. For a triangle *K* with barycenter ${\textbf{M}}_{K}$ we introduce a neighborhood in terms of some given $\alpha \in [0,2\pi [$ and a scaling parameter $\lambda \ge 0$ below. Consider the infinite line

A.1 $$\begin{aligned} L_{\alpha }:=\left\{ {\textbf{M}}_{K}+s\left( {\begin{array}{c}\cos \alpha \\ \sin \alpha \end{array}}\right) :s\in {\mathbb {R}}\right\} \text {.} \end{aligned}$$

and let $s_{+}>0$ be such that $${\textbf{y}}_{\alpha }:={\textbf{M}}_{K}+s_{+}\left( {\begin{array}{c}\cos \alpha \\ \sin \alpha \end{array}}\right) \in L_{\alpha }\cap \partial K$$. Denote the midpoint of the intersection $L_{\alpha }\cap K$ by ${\textbf{M}}_{\alpha }\in K$. Given $\lambda \ge 0$, the neighbourhood reads

A.2 $$\begin{aligned} K_{\lambda }:=\left\{ {\textbf{M}}_{\alpha }+s\left( {\textbf{y}}_{\alpha }-{\textbf{M}}_{\alpha }\right) \mid \left\{ \begin{array}{l} 0\le s\le 1+\lambda \\ 0\le \alpha <2\pi \end{array} \right. \quad \right\} . \end{aligned}$$

Clearly $$K=K_{0}\subseteq K_{\lambda }$$ for $$\lambda \ge 0$$.

### Lemma A.1

There exists $c>0$ depending solely on the shape regularity of *K* such that for any $\lambda \ge 0:$

A.3 $$\begin{aligned} \left\{ {\textbf{y}}\in {\mathbb {R}}^{2}\backslash K\mid c\frac{{\text {dist}}\left( {\textbf{y}},K\right) }{h_{K}}\le \lambda \right\} \subset K_{\lambda }\quad \text {and\quad }{\text {diam}}K_{\lambda } \le \left( 1+2\lambda \right) h_{K}. \end{aligned}$$

### Proof

Since $K_{\lambda }$ is compact, there exist ${\textbf{y}},{\textbf{z}}\in \partial K_{\lambda }$ such that

A.4 $$\begin{aligned} {\text {diam}}K_{\lambda }=\left\| {\textbf{y}}-{\textbf{z}}\right\| . \end{aligned}$$

From (A.2) it follows that there exist $0\le \alpha ,\beta <2\pi$ such that

$$\begin{aligned} {\textbf{y}}={\textbf{M}}_{\alpha }+\left( 1+\lambda \right) \left( {\textbf{y}}_{\alpha }-{\textbf{M}}_{\alpha }\right) \quad \text {and}\quad {\textbf{z}}={\textbf{M}}_{\beta }+\left( 1+\lambda \right) \left( {\textbf{z}} _{\beta }-{\textbf{M}}_{\beta }\right) . \end{aligned}$$

Rearranging both terms yields

A.5 $$\begin{aligned} {\textbf{y}}={\textbf{y}}_{\alpha }+\lambda \left( {\textbf{y}}_{\alpha } -{\textbf{M}}_{\alpha }\right) \quad \text {and}\quad {\textbf{z}}={\textbf{z}}_{\beta }+\lambda \left( {\textbf{z}}_{\beta }-{\textbf{M}}_{\beta }\right) . \end{aligned}$$

Two triangle inequalities and (A.4)–(A.5) result in

$$\begin{aligned} {\text {diam}}K_{\lambda }&\le \left\| {\textbf{y}}-{\textbf{y}} _{\alpha }\right\| +\left\| \mathbf {y_{\alpha }-{\textbf{z}}_{\beta } }\right\| +\left\| {\textbf{z}}-{\textbf{z}}_{\beta }\right\| \le h_{K}+\lambda \left( \left\| {\textbf{y}}_{\alpha }-{\textbf{M}}_{\alpha }\right\| +\left\| {\textbf{z}}_{\beta }-{\textbf{M}}_{\beta }\right\| \right) \\&\le h_{K}+2\lambda \sup _{0\le \gamma <2\pi }\left\| {\textbf{y}}_{\gamma }-{\textbf{M}}_{\gamma }\right\| \le \left( 1+2\lambda \right) h_{K}. \end{aligned}$$

To prove the first inclusion in (A.3), let ${\textbf{y}}\in {\mathbb {R}}^{2}\backslash K$ and $\alpha \in [ 0,2\pi [$, $s\ge 0$ such that

A.6 $$\begin{aligned} {\textbf{y}}={\textbf{M}}_{\alpha }+\left( 1+s\right) \left( {\textbf{y}}_{\alpha }-{\textbf{M}}_{\alpha }\right) . \end{aligned}$$

Rearranging the terms as in (A.5) implies that

$$\begin{aligned} \left\| {\textbf{y}}-{\textbf{y}}_{\alpha }\right\| =s\left\| {\textbf{y}}_{\alpha }-{\textbf{M}}_{\alpha }\right\| . \end{aligned}$$

Elementary trigonometry provides a constant $0<c$ depending only on the shape regularity of *K* such that $\left\| {\textbf{y}}-{\textbf{y}}_{\alpha }\right\| \le \sqrt{c}{\text {dist}}\left( {\textbf{y}},K\right)$ and $\left\| {\textbf{y}}_{\alpha }-{\textbf{M}}_{\alpha }\right\| \ge h_{K}/\sqrt{c}$ so that

$$\begin{aligned} s=\frac{\left\| {\textbf{y}}-{\textbf{y}}_{\alpha }\right\| }{\left\| {\textbf{y}}_{\alpha }-{\textbf{M}}_{\alpha }\right\| }\le c \frac{{\text {dist}}\left( {\textbf{y}},K\right) }{h_{K}}. \end{aligned}$$

If the right-hand side is bounded by $\lambda$, then $s\le \lambda$ follows and (A.6) implies ${\textbf{y}}\in K_{\lambda }$. This concludes the proof. $\square$

### Lemma A.2

Given a triangle *K*, there exists $c\ge 1$ depending only on the shape regularity of *K* such that any polynomial $p\in {\mathbb {P}}_{k}\left( {\mathbb {R}}^{2}\right)$ satisfies

A.7 $$\begin{aligned} \left| p\left( {\textbf{y}}\right) \right| \le \left| T_{k}\left( 1+\lambda \right) \right| \left\| p\right\| _{L^{\infty }\left( K\right) }\quad \forall {\textbf{y}}\in {\mathbb {R}}^{2}\backslash K\quad \text {for }\lambda :=c\frac{{\text {dist}}\left( {\textbf{y}},K\right) }{h_{K}}. \end{aligned}$$

The Chebyshev polynomial $T_{k}$ outside the interval $\left[ -1,1\right]$ admits

A.8 $$\begin{aligned} \left| T_{k}\left( 1+\lambda \right) \right| \le \gamma _{k}\left( \lambda \right) :=\min \left\{ \frac{2^{k}+1}{2}\left( 1+\lambda \right) ^{k},{\text {e}}^{\lambda k^{2}/2}\right\} . \end{aligned}$$

### Proof

We start with a one-dimensional consideration. It is well known (see, e.g., [18, Prop. 2.3, 2.4]) that any polynomial $f\in {\mathbb {P}}_{k}\left( {\mathbb {R}}\right)$ satisfies

$$\begin{aligned} \left| f\left( y\right) \right| \le \left\| f\right\| _{L^{\infty }\left( \left[ -1,1\right] \right) }\max \left\{ 1,\left| T_{k}\left( y\right) \right| \right\} \quad \forall y\in {\mathbb {R}}. \end{aligned}$$

An affine transformation to the interval $I=\left[ m-\varepsilon ,m+\varepsilon \right]$ for $m\in {\mathbb {R}}$ and $\varepsilon >0$ provides

A.9 $$\begin{aligned} \left| f\left( y\right) \right| \le \left\| f\right\| _{L^{\infty }\left( I\right) }\max \left\{ 1,\left| T_{k}\left( \frac{y-m}{\varepsilon }\right) \right| \right\} \quad \forall y\in {\mathbb {R}}. \end{aligned}$$

Next, consider $p\in {\mathbb {P}}_{k}\left( {\mathbb {R}}^{2}\right)$. Any ${\textbf{y}}\in K_{\lambda }\backslash K$ fulfills ${\textbf{y}}={\textbf{M}}_{\alpha }+\left( 1+s\right) \left( {\textbf{y}}_{\alpha }-{\textbf{M}}_{\alpha }\right)$ for some $\alpha \in [ 0,2\pi [$, $0\le s\le \lambda$ with ${\textbf{y}}_{\alpha }\in \partial K$ and ${\textbf{M}}_{\alpha }\in K$. We choose a coordinate system such that ${\textbf{M}}_{\alpha }$ is the origin and ${\textbf{y}}=\left( y_{1},0\right) ^{T}$ for some $y_{1}>0$ and set $\rho :=\left\| {\textbf{y}}_{\alpha }-{\textbf{M}}_{\alpha }\right\|$. Estimate (A.9) applies to $f=p|_{y_2=0}\in {\mathbb {P}}_k({\mathbb {R}})$ for the interval $\left[ -\rho ,\rho \right] \times \left\{ 0\right\} \subset K$ with $y_1-m=\left\| {{\textbf {y}}}-{{\textbf {M}}}_{\alpha }\right\| =\left( 1+s\right) \rho$. Since $s\ge 0$, it follows that

$$\begin{aligned} \left| p\left( {\textbf{y}}\right) \right| \le \left\| p\right\| _{L^{\infty }\left( K\right) }T_{k}\left( 1+s\right) \le \left\| p\right\| _{L^{\infty }\left( K\right) }T_{k}\left( 1+\lambda \right) . \end{aligned}$$

Finally, we prove (A.8). For $y\in {\mathbb {R}}$ with $\left| y\right| \ge 1$, the Chebyshev polynomials have the representation (see, e.g., [18, Prop. 2.5])

$$\begin{aligned} T_{k}\left( y\right) =\frac{\left( y+\sqrt{y^{2}-1}\right) ^{k}+\left( y-\sqrt{y^{2}-1}\right) ^{k}}{2}. \end{aligned}$$

From this $\left| T_{k}\left( y\right) \right| \le \left( 2^k +1\right) /2\left| y\right| ^{k}$ follows directly for $\left| y\right| \ge 1$. Hence,

A.10 $$\begin{aligned} \left| p\left( {\textbf{y}}\right) \right| \le \left\| p\right\| _{L^{\infty }\left( K\right) }\frac{2^{k}+1}{2}\left( 1+\lambda \right) ^{k}\quad \forall {\textbf{y}}\in K_{\lambda }. \end{aligned}$$

If ${\textbf{y}}$ is close to *K*, this estimate can be improved. From [14, Lem. A.1] we conclude that

$$\begin{aligned} \left| T_{k}\left( y\right) \right| \le {\text {e}}^{\left( \left| y\right| -1\right) k^{2}/2}\quad \text {for }\left| y\right| \ge 1. \end{aligned}$$

Consequently, we have

$$\begin{aligned} \left| p\left( {\textbf{y}}\right) \right| \le \left\| p\right\| _{L^{\infty }\left( K\right) }{\text {e}}^{\lambda k^{2} /2}\quad \forall {\textbf{y}}\in K_{\lambda }. \end{aligned}$$

From Lemma A.1, the estimate (A.7) for the relevant values of ${\textbf{y}}$ follows. $\square$

The next lemma is concerned with a neighborhood property for polynomial approximations.

### Lemma A.3

Let $p\in H^{s-1}\left( {\mathbb {R}}^{2}\right)$ for some $s>1$. Given two triangles $K, K^*$, let $\delta \ge 0$ satisfy

$$\begin{aligned} K\subset K^{*}\subset \left\{ {\textbf{y}}\in {\mathbb {R}}^{2}\mid {\text {dist}}\left( {\textbf{y}},K\right) \le \delta h_{K}\right\} . \end{aligned}$$

Then, for any polynomial $p_{k}\in {\mathbb {P}}_{k}\left( {\mathbb {R}}^{2}\right)$, it holds

$$\begin{aligned} \left\| p-p_{k}\right\| _{L^{2}\left( K^{*}\right) }\le T_{k}\left( 1+c\delta \right) \left( 2\frac{\left( \left( 1+2\delta \right) h_{K}\right) ^{\min \left\{ k+1,s-1\right\} }}{\left( k+1\right) ^{s-1}}\left\| p\right\| _{H^{s-1}\left( K^{*}\right) }+\left\| p-p_{k}\right\| _{L^{2}\left( K\right) }\right) , \end{aligned}$$

where *c* and *C* only depend on the shape regularity of *K* and $K^{*}$. The Chebyshev polynomial $T_{k}$ can be estimated by $\gamma _{k}\left( c\delta \right)$ with $\gamma _{k}$ as in (A.8).

### Proof

We employ the operator $W_{k}:H^{s-1}\left( K^{*}\right) \rightarrow {\mathbb {P}}_{k}\left( K^{*}\right)$ from [1, Cor. 3.2] based on the operator in [23, Lem. 3.1] to obtain

A.11 $$\begin{aligned} \left\| p-W_{k}p\right\| _{L^{2}\left( K^{*}\right) }\le C\frac{\left( \left( 1+2\delta \right) h_{K}\right) ^{\min \left\{ k+1,s-1\right\} }}{\left( k+1\right) ^{s-1}}\left\| p\right\| _{H^{s-1}\left( K^{*}\right) }. \end{aligned}$$

A triangle inequality leads to

$$\begin{aligned} \left\| p-p_{k}\right\| _{L^{2}\left( K^{*}\right) }\le \left\| p-W_{k}p\right\| _{L^{2}\left( K^{*}\right) }+\left\| W _{k}p-p_{k}\right\| _{L^{2}\left( K^{*}\right) }. \end{aligned}$$

For any $p_k\in {\mathbb {P}}_k(K^*)$, Lemma A.2 with $\lambda =c\delta$ provides

$$\begin{aligned} \left\| W_{k}p-p_{k}\right\| _{L^{2}\left( K^{*}\right) }&=T_{k}\left( 1+\lambda \right) \left\| \left( W_{k}p-p_{k}\right) \right\| _{L^{2}\left( K\right) }\\&\le T_{k}\left( 1+\lambda \right) \left( \left\| p-W_{k} p\right\| _{L^{2}\left( K^{*}\right) }+\left\| p-p_{k}\right\| _{L^{2}\left( K\right) }\right) , \end{aligned}$$

with a triangle inequality in the last step. This and a triangle inequality lead to

$$\begin{aligned} \left\| p-p_{k}\right\| _{L^{2}\left( K^{*}\right) }\le \left( 1+T_{k}\left( 1+\lambda \right) \right) \left\| p-W_{k}p\right\| _{L^{2}\left( K^{*}\right) }+T_{k}\left( 1+\lambda \right) \left\| p-p_{k}\right\| _{L^{2}\left( K\right) }. \end{aligned}$$

Hence, the claim follows from (A.11) and $1\le T_{k}\left( 1+\lambda \right)$. $\square$
